# Clathrin-associated SCYL2 contributes to the activation of PI3K/AKT signaling and tumorigenesis through PTEN phosphorylation in adult T-cell leukemia/lymphoma

**DOI:** 10.1038/s41417-026-01008-9

**Published:** 2026-02-16

**Authors:** Tomonaga Ichikawa, Shunsuke Shimosaki, Shingo Nakahata, Akira Suekane, Issay Kitabayashi, Hidekatsu Iha, Kazuya Shimoda, Takashi Murakami, Kazuhiro Morishita

**Affiliations:** 1 Department of Microbiology, Saitama Medical University, Saitama, Japan; 2 Division of Tumor and Cellular Biochemistry, Department of Medical Sciences, University of Miyazaki, Miyazaki, Japan; 3 Division of HTLV-1/ATL Carcinogenesis and Therapeutics, Joint Research Center for Human Retrovirus Infection, Kagoshima University, Kagoshima, Japan; 4 Department of Acute Critical Care and Disaster Medicine, Institute of Science Tokyo Graduate School of Medical and Dental Sciences, Institute of Science Tokyo, Tokyo, Japan; 5 Oncology Innovation Center/ Center for Translational Research, Fujita Health University, Aichi, Japan; 6 Division of Pathophysiology, The Research Center for GLOBAL and LOCAL Infectious Diseases (RCGLID), Oita University, Yufu, Oita, Japan; 7 Division of Hematology, Diabetes, and Endocrinology, Department of Internal Medicine, Faculty of Medicine, University of Miyazaki, Miyazaki, Japan; 8 Division of Pediatrics, Developmental and Urological-Reproductive Medicine, Faculty of Medicine, University of Miyazaki, Miyazaki, Japan

**Keywords:** Leukaemia, Cell biology

## Abstract

Inactivation of PTEN by post-translational modifications causes aberrant amplification of the PI3K/AKT signaling pathway in many tumors. PTEN is a tumor suppressor phosphatase that is frequently phosphorylated at conserved serine/threonine residues (S380, T382, and T383 clusters) in the C-terminal tail of ATL and various solid cancer cells. Here, we identify SCY1-like protein 2 (SCYL2), with a protein kinase-like domain, as a novel PTEN-binding protein; however, the mechanism by which SCYL2 regulates PTEN phosphorylation remains unclear. SCYL2-associated complex phosphorylates PTEN at STT, and SCYL2 downregulation has anti-tumor effects in ATL via inhibition of the PI3K/AKT signaling pathway by dephosphorylating PTEN at STT. SCYL2 reportedly binds to the clathrin heavy chain (CHC), which regulates cytoplasmic vesicle formation, trafficking, and signaling pathways. Our results indicate that SCYL2 expression induces the binding of CHC to PTEN. Furthermore, the inhibition of clathrin-coated vesicles (CCVs) by CHC downregulation or inhibition suppresses cell survival by reducing phosphorylated PTEN at the STT, suggesting that SCYL2 enhances PTEN phosphorylation through CCVs as a signaling platform. Our results indicate that SCYL2/CHC complex plays a pivotal role in regulating the PI3K/AKT signaling pathway through PTEN phosphorylation, thus leading to tumor development and may be a promising novel target for treating tumors.

## Introduction

Human T-cell leukemia virus type 1 (HTLV-1), an oncogenic retrovirus, is associated with an aggressive malignant disease involving a cluster of differentiation (CD)4^+^ T lymphocytes, known as adult T-cell leukemia/lymphoma (ATL) [1, 2]. Recent studies have demonstrated that constitutive activation of signal transduction pathways such as phosphoinositide 3-kinase (PI3K)/protein kinase B (AKT) via inactivation of phosphatase and tensin homolog deleted on chromosome 10 (PTEN) plays a vital role in the development of ATL and other cancers [3, 4].

The tumor suppressor PTEN is a lipid phosphatase that dephosphorylates the lipid second messenger phosphatidylinositol 3,4,5-trisphosphate (PIP3) to PI(4,5)P2 in the cell membrane. Because PI3K catalyzes PIP3 production, which then activates several downstream kinases, including AKT, PTEN regulates cell proliferation, inflammation, and migration by opposing the PI3K/AKT signaling pathway [5, 6]. Although *PTEN* is reportedly lost or mutated in a wide range of human cancers, leading to aberrant activation of the PI3K/AKT signaling pathway, PTEN expression is retained in most cancers, despite PTEN inactivation [7, 8]. Furthermore, the catalytic activation of PTEN is regulated by post-translational modifications, and its inactivation is characterized by serine/threonine phosphorylation [9, 10]. On the other hand, PTEN phosphorylation at tyrosine affects activity and stability [11]. Structurally, PTEN consists of an N-terminal phosphatase domain, C2 domain, PDZ-binding motif, and regulatory C-terminal tail (C-tail). Phosphorylation of a cluster of serine (S) and threonine (T) residues at S370, S380, T382, T383, and S385 in the C-tail of PTEN affects phosphatase activity, protein stability, and cellular localization. High phosphorylation of the cluster of S/T residues at the C-tail occurs in many types of cancer via disruption of the balance between PTEN kinase and phosphatase [12, 13]. We previously identified N-myc downstream-regulated gene 2 (NDRG2) as a PTEN-binding protein that recruits protein phosphatase 2A (PP2A) to promote PTEN dephosphorylation at S380, T382, and T383 (STT) clusters. Our results indicate that NDRG/PP2A complex works as a PTEN protein phosphatase, and PTEN (STT) phosphorylation (but not S370 and S385) plays an important role for tumorigenesis in ATL and other cancers [4, 14–17]. Although casein kinase 2 (CK2) has been identified as a candidate PTEN protein kinase for phosphorylation at the C-tail [18, 19], the precise molecular regulatory mechanisms underlying PTEN phosphorylation have not yet been elucidated.

To identify a protein kinase for PTEN in ATL, we profiled the proteins that interacted with PTEN. Thus, we identified a novel PTEN-binding protein, SCY1-like protein 2 (SCYL2), which has an N-terminal protein pseudokinase domain that co-localizes with the clathrin heavy chain (CHC), mainly in the cytoplasm of ATL cells. SCYL2 functions as a component of clathrin-coated vesicles (CCVs) through phosphorylation of the β2 subunit of the plasma membrane adaptor protein 2 complex (AP2) and regulates trans-Golgi networks, HIV-1 release, and frizzled-5 lysosomal degradation in a manner dependent or independent of CHC binding [20–24]. In *SCYL2*-deficient mice, although surviving animals exhibit severe neurological disorders of CA3 pyramidal neurons [25], the detailed mechanisms responsible for tumorigenesis remain poorly understood. CHC expression is frequently upregulated in various types of cancer and is a valuable marker for tumor diagnosis [26, 27]. Signaling from plasma membrane receptors and molecules is internalized and transferred to early endosomes with CCVs as cargo. This leads to internalization, sorting into degradation or recycling, and endosomes as mobile signaling platforms. This results in the activation of signaling pathways by cytoplasmic vesicles or endosomes, and many proteins form signaling assemblies via clathrin-mediated endocytosis [28, 29].

In the present study, we found that SCYL2 was highly expressed in ATL cells. The N-terminal kinase-like domain of SCYL2 was demonstrated to be essential for PTEN binding, and SCYL2-associated complex phosphorylated PTEN at the STT. The presence of SCYL2 enhanced CHC binding to PTEN, and inhibition of SCYL2 or CHC expression by specific short hairpin RNA (shRNA)-induced knockdown or treatment with the CCVs inhibitor chlorpromazine (CPZ) suppressed cell viability and activation of the PI3K/AKT signaling pathway through the dephosphorylation of PTEN at STT in ATL cells. Therefore, we propose that PTEN in the STT cluster is phosphorylated by SCYL2/CHC-induced vesicles as a mobile signaling platform followed by the enhancement and extension of PI3K/AKT signaling pathway, and that SCYL2/CHC inhibition may be a promising novel target for the diagnosis and therapy of ATL.

## Results

### SCYL2 is a novel PTEN-binding protein

**Fig. 1:**
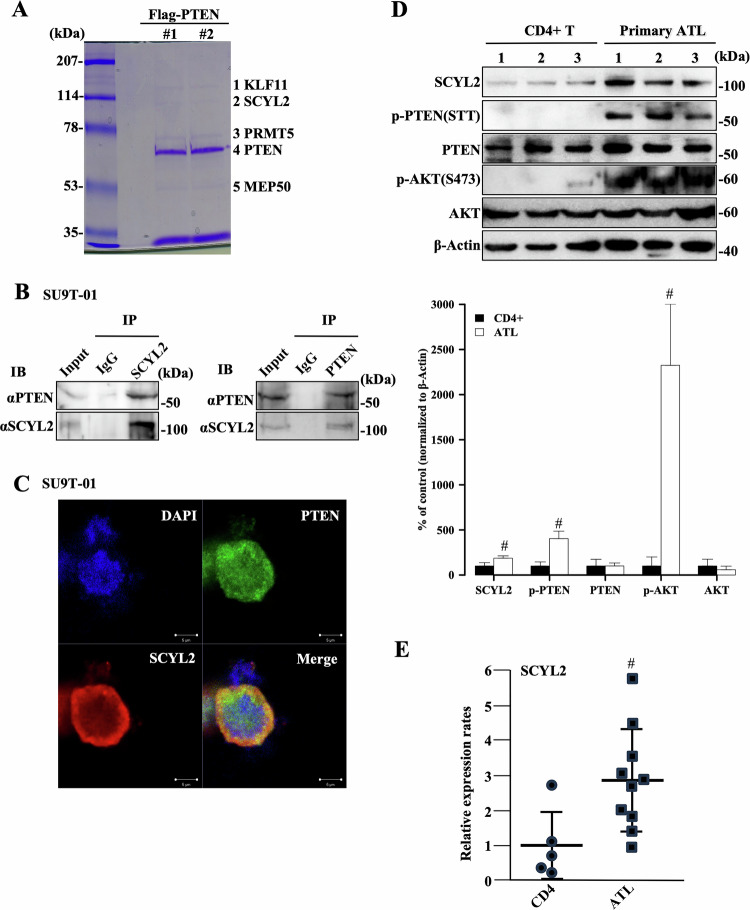
SCYL2 is a novel PTEN-binding protein. **A** Co-immunoprecipitated anti-Flag antibody for PTEN (lanes 1 and 2) is shown in an image of Coomassie brilliant blue-stained SDS-PAGE gel after transfection of the Flag-PTEN into the ATL cell line KK1. The in-gel tryptic cleavage products of each major band (1–5) were also sequenced by MS (Supplementary Table 1). Molecular weights are indicated in kDa. **B** Cell lysates from the ATL cell line SU9T-01 were precipitated using anti-SCYL2 or anti-PTEN antibodies, and the precipitated proteins were immunoblotted with each specific antibody. **C** PTEN (Alexa Fluor-488, green) and SCYL2 (Alexa Fluor-555, red) were detected in SU9T-01 cells by immunofluorescent staining, and nuclei were stained with DAPI (blue). Scale bar, 5 µm. **D** Western blot analysis of SCYL2, phosphorylated (p)-PTEN (STT), PTEN, p-AKT (S473), and AKT in CD4^+^ T lymphocytes from healthy volunteers (CD4^+^, used as controls) and primary ATL cells from patients with acute-type ATL. Bar graphs show quantification of the relative band intensity normalized to β-actin. The mean and SD are shown (*n* = 3); #*p* < 0.05, versus CD4^+^. **E** qPCR analysis of SCYL2 mRNA in CD4^+^ cells (*n* = 5) and primary ATL cells (*n* = 10). Data are presented as a dot plot and are shown as the mean ± SD; #*p* < 0.05, versus CD4^+^.

To isolate PTEN-associated proteins, the Flag-PTEN was introduced into the ATL cell line KK1, and the PTEN-binding proteins were precipitated with an anti-Flag antibody for comprehensive profiling using Mass spectrometry (MS). We isolated several candidate PTEN-binding proteins, including SCYL2 with a protein kinase-like domain (Fig. 1A and Supplementary Table 1). To confirm our MS results, we used immunoprecipitation to examine the interaction between SCYL2 and PTEN in ATL cell lines. Endogenous PTEN co-precipitated with endogenous SCYL2. Reciprocally, SCYL2 was pulled down in ATL cells when antibodies against endogenous PTEN were used for immunoprecipitation (Fig. 1B and Supplementary Fig. 1A). Furthermore, immunofluorescence revealed that PTEN and SCYL2 were mainly colocalized in the cytoplasm and perinuclear region of ATL cells (Fig. 1C and Supplementary Fig. 1B). These results indicated that endogenous SCYL2 interacts with PTEN in the cytoplasm of ATL cells.

To analyze the expression of SCYL2 in patient-derived primary ATL cells and ATL cell lines, we used published microarray gene expression data (GSE33615, GSE55851, and GSE43017) from patients with acute ATL versus CD4^+^ T cells or PBMCs from healthy donors [4, 30, 31]. Although SCYL2 expression did not change in HTLV-1-infected CD4^+^ T cells from acute ATL patients compared to CD4^+^ T cells from healthy donors (GSE33615), cells from acute ATL patients with cell adhesion molecule 1 (CADM1)^+^/CD7^−^ as a surrogate marker of progression had significantly higher SCYL2 expression than PBMCs from healthy donors (GSE55851) (Supplementary Fig. 1C). The gene expression profiles of acute-type ATL and CD4^+^ T cells revealed that SCYL2 expression was significantly upregulated at the mRNA and protein levels in primary ATL cells compared with CD4^+^ T cells, and the phosphorylation of AKT at S473 and PTEN at STT was detected in most primary ATL cells (Fig. 1D, E, and Supplementary Fig. 1C). Furthermore, SCYL2 expression was remarkably higher in HTLV-1-infected (MT2 and HUT102) and ATL (KOB, KK1, and SU9T-01) cell lines than in HTLV-1-negative T-ALL (Jurkat and MOLT4) cell lines (Supplementary Fig. 1D). It is possible that upregulated SCYL2 binds to PTEN and regulates the PI3K/AKT signaling pathway in ATL.

### SCYL2-associated complex regulates PTEN phosphorylation at STT

**Fig. 2:**
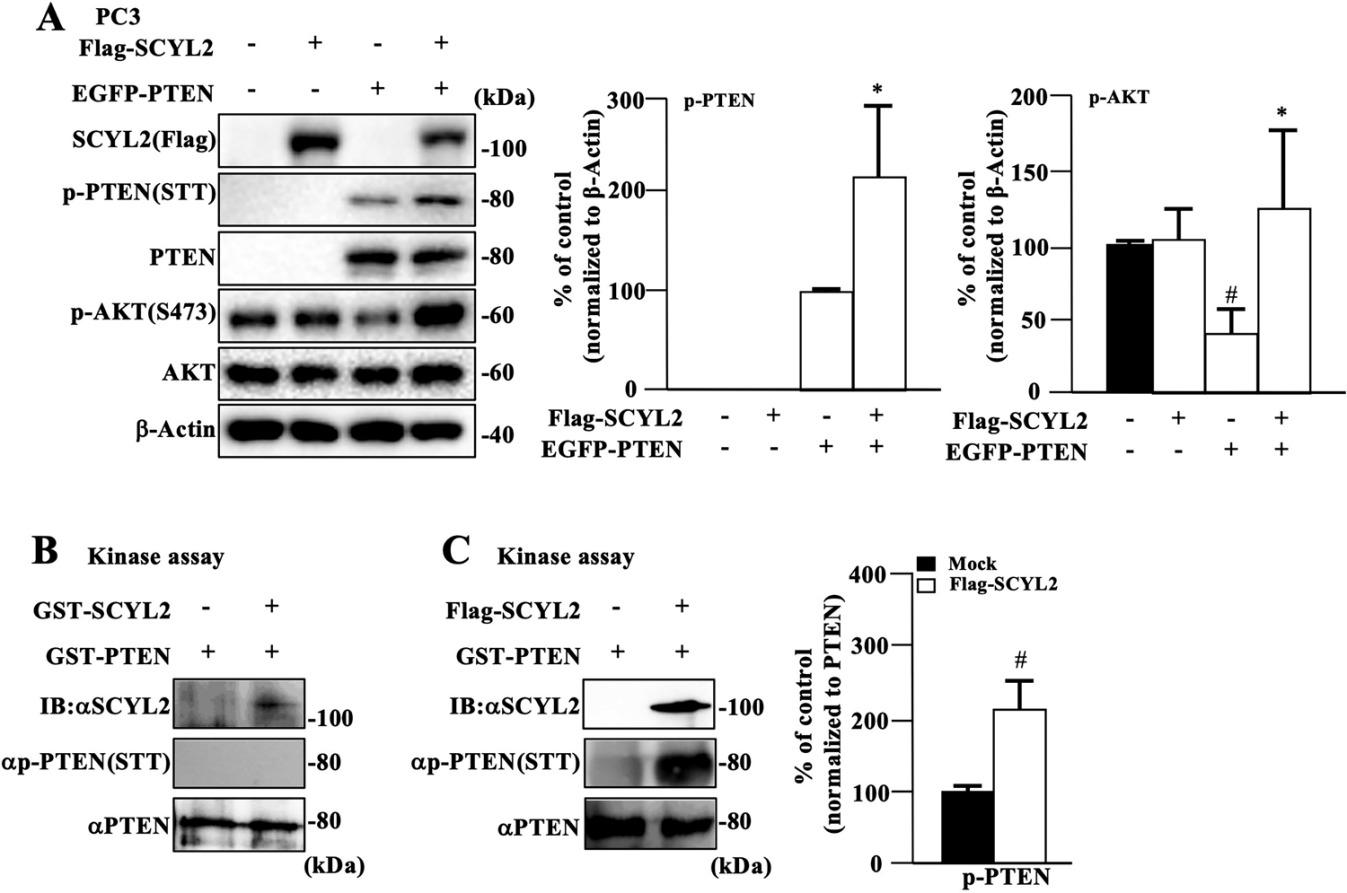
SCYL2-associated complex regulates PTEN phosphorylation at STT. **A** PTEN-deficient prostate cancer PC3 cells were co-transfected with a Flag-SCYL2 and EGFP-PTEN, and whole lysates were probed for the indicated antibodies. The results are representative of three independent experiments. Bar graphs show the quantification of relative band intensity normalized to β-actin. The mean and SD are shown (*n* = 3); #*p* < 0.05, versus −/−; *p < 0.05, versus −/+. **B** Kinase activity of GST-SCYL2 measured using an in vitro kinase assay with GST-PTEN as the substrate. Kinase activity was determined by immunoblotting using an anti-p-PTEN (STT) antibody. The results are representative of three independent experiments. **C** Kinase activity of immunoprecipitates from 293T cells transfected with Flag-SCYL2 using anti-Flag antibody, measured using an in vitro kinase assay with GST-PTEN as a substrate. The results are representative of three independent experiments. Bar graphs show the quantification of the relative band intensity normalized to PTEN. The mean and SD are shown (*n* = 3); #*p* < 0.05, versus mock.

We examined the involvement of SCYL2 in regulating the PI3K/AKT signaling pathway. The dose-dependent transfection of Flag-SCYL2 into 293T cells revealed a marked induction of AKT phosphorylation at S473 and PTEN phosphorylation at STT. In contrast, PTEN phosphorylation at S370 and S385 did not change remarkably (Supplementary Fig. 2A). To determine whether SCYL2 directly affects AKT phosphorylation in the absence of PTEN expression, we co-transfected Flag-SCYL2 with or without EGFP-PTEN in PTEN-deficient PC3 cells and examined the phosphorylation status of AKT. Although SCYL2 expression alone did not enhance AKT phosphorylation, PTEN expression induced the suppression of AKT phosphorylation in PC3 cells. Furthermore, co-transfection of PC3 cells with Flag-SCYL2 and EGFP-PTEN restored the phosphorylation status of AKT along with high PTEN phosphorylation, indicating that SCYL2 regulates AKT activation through PTEN phosphorylation at STT (Fig. 2A).

Next, we explored which domain of SCYL2 was directly associated with PTEN. Three EGFP-deletion mutants of SCYL2 (1–697, 1–375, or 699–929), and full-length SCYL2 (WT) (Supplementary Fig. 2B) were separately co-transfected with a Flag-PTEN into 293T cells. PTEN co-precipitated with SCYL2/WT, SCYL2/1–697, or SCYL2/1–375 (kinase domain) but only weakly co-precipitated with SCYL2/699–929 (clathrin binding) (Supplementary Fig. 2C). We also generated two EGFP-PTEN deletion mutants (N with PTPase or C with C2 and C-tail) and one with full-length PTEN (WT) (Supplementary Fig. 2B). Flag-SCYL2 co-precipitated with PTEN/WT or PTEN/C and was greatly reduced with PTEN/N (Supplementary Fig. 2D), indicating that the kinase domain of SCYL2 is involved in the interaction with the C2/C-tail of PTEN.

An in vitro kinase assay was performed to determine whether SCYL2 phosphorylated PTEN at the STT, as previously described [32]. We used GST-PTEN as a substrate and GST-SCYL2 as a kinase. GST-SCYL2 could not enhance phosphorylation of PTEN at the STT in vitro (Fig. 2B). In the next experiment, the kinase activity of SCYL2 purified with an anti-Flag antibody was analyzed by in vitro kinase assay using GST-PTEN as a substrate. Flag-SCYL2 immunoprecipitated from transfected 293T cells significantly induced PTEN phosphorylation at the STT in vitro (Fig. 2C). These findings suggest that SCYL2-binding proteins may regulate PTEN phosphorylation at STT, leading to the activation of the PI3K/AKT pathway.

### SCYL2 plays a vital role in tumorigenesis by regulating the PI3K/AKT signaling pathway in ATL in vitro and in vivo

**Fig. 3:**
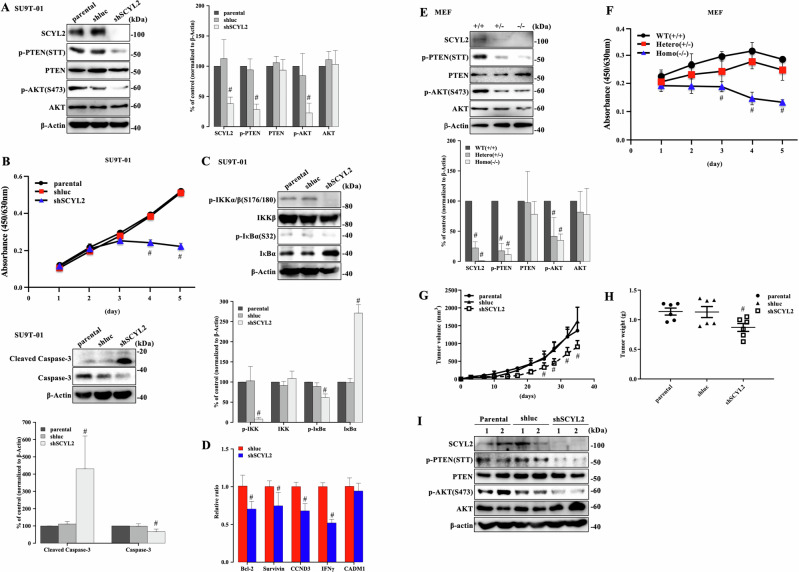
SCYL2 plays a vital role in tumorigenesis by regulating the PI3K/AKT signaling pathway in ATL in vitro and in vivo*.* **A** Cell lysate from SU9T-01 cells (parental, shluc, and shSCYL2) was investigated using antibodies specific to each immunoblot. The results are representative of three independent experiments. Bar graphs show the quantification of relative band intensity normalized to β-actin. The mean and SD are shown (*n* = 3); #*p* < 0.05, versus parental. **B** Cell growth curves of SU9T-01 cells for 5 days. The mean and SD are shown (*n* = 4); #*p* < 0.05, versus parental. The cell apoptosis (Cleaved Caspase-3 and Caspase-3) was investigated using each specific antibody in immunoblots. The results are representative of three independent experiments. Bar graphs show the quantification of relative band intensity normalized to β-actin. The mean and SD are shown (*n* = 3); #*p* < 0.05, versus parental. **C** Cell lysate from SU9T-01 cells (parental, shluc, and shSCYL2) was subjected to western blot analysis of NF-κB pathway. The results are representative of three independent experiments. Bar graphs show the quantification of relative band intensity normalized to β-actin. The mean and SD are shown (*n* = 3); #*p* < 0.05, versus parental. **D** Quantitative PCR analysis of NF-κB target genes in SU9T-01 cells (shluc and shSCYL2). The mean and SD are shown (*n* = 4); #*p* < 0.05, versus shluc. **E** Cell lysate form *SCYL2* WT (+/+), hetero (+/−), and homo (−/−) MEF was investigated using specific antibodies in immunoblots. The results are representative of three independent experiments. Bar graphs show the quantification of the relative band intensity normalized to β-actin. The mean and SD are shown (*n* = 3); #*p* < 0.05, versus WT (+/+). **F** Cell growth curves of MEF after 5 days. The mean and SD are shown (*n* = 4); #*p* < 0.05, versus WT (+/+). **G** SU9T-01 cells were injected subcutaneously into NOG mice (*n* = 6) and the tumors were measured on the indicated days. The mean and SD are shown (*n* = 6); #*p* < 0.05, versus parental. **H** Tumor weight was measured after mice were sacrificed. The mean and SD are shown (*n* = 6); #*p* < 0.05, versus parental. **I** Cell lysate from tumors developed in NOG mice was investigated using antibodies specific to each immunoblot.

We investigated whether SCYL2 expression was associated with cell viability through PTEN (STT) phosphorylation in ATL cells. SCYL2 expression was suppressed by shRNA with three different sequences against SCYL2 (shSCYL2-1, -2, and -3) compared to parental and shluc control ATL (SU9T-01 and KK1) cells. SCYL2 knockdown in ATL cell lines predominantly suppressed SCYL2 expression compared to parental and shluc ATL cell lines, followed by a decrease in phosphorylated AKT and PTEN (Supplementary Fig. 3A). Therefore, we selected shSCYL2-1 to examine phosphorylation levels and cell viability. AKT and PTEN phosphorylation were significantly lower in ATL cell lines with SCYL2 knockdown (shSCYL2-1) than in parental and shluc ATL cell lines (Fig. 3A and Supplementary Fig. 3B). In addition, to investigate whether PTEN lipid phosphatase activity is regulated by SCYL2 expression, PTEN were immunoprecipitated from SU9T-01 and KK1 cells (shluc and shSCYL2) and were measured for an in vitro phosphatase assay using phosphatidylinositol 3,4,5-trisphosphate diC8 (PIP3 diC8) as PIP3 substrate, as previously described [33, 34]. PTEN immunoprecipitated from cell lysate of shSCYL2 ATL cells significantly dephosphorylated PIP3 compared to shluc (Supplementary Fig. 3C), indicating that PTEN lipid phosphatase activity is increased by the suppression of PTEN phosphorylation via SCYL2 knockdown leading to the inhibition of AKT phosphorylation. Furthermore, the downregulation of SCYL2 expression suppressed the cell viability rate through the induction of Cleaved Caspase-3 as a marker of cell apoptosis in ATL cells (Fig. 3B and Supplementary Fig. 3D). We identified that nuclear factor kappaB (NF-κB) signaling pathway is constitutively activated in ATL cells through the phosphorylation of IKK by AKT activation, playing an essential role in the pathogenesis of ATL [1, 17, 35, 36]. The downregulation of SCYL2 expression significantly suppressed the phosphorylation of IKKα/β/IκBα and degradation of IκBα resulting in the inhibition of NF-κB target genes in ATL cells (Fig. 3C, D, and Supplementary Fig. 3E, F). Furthermore, we performed reverse experiments by stably introducing the Flag-SCYL2 expression vector into U2OS and HeLa cell lines, which have low endogenous SCYL2 expression. Although PTEN phosphorylation at S370 and S385 was not altered, AKT phosphorylation and PTEN (STT) phosphorylation were markedly increased in SCYL2-enhanced cell lines, which was accompanied by an increase in the cell viability compared with the mock control (Supplementary Fig. 3G, H).

To determine whether SCYL2 plays a physiological role in cell viability in vivo, we generated *SCYL2*-deficient mice (Supplementary Fig. 3I, J), and all *SCYL2* homozygous (homo, −/−) mice died soon after birth. Therefore, we established mouse MEF from *SCYL2*-deficient (−/− and +/−) and WT (+/+) mice and analyzed the activation of the PI3K/AKT signaling pathway and cell viability. *SCYL2* (−/−) and (+/−) MEF exhibited decreased phosphorylation of AKT at S473 and PTEN at STT and decreased cell viability compared to WT (+/+) MEF (Fig. 3E, F).

To investigate the effects of SCYL2 on ATL cell tumor growth in vivo, parental, shluc, and shSCYL2-transfected SU9T-01 cells were subcutaneously implanted into immunodeficient NOG mice. Compared to SU9T-01-parental and -shluc cells, xenograft volume and weight were significantly suppressed in SU9T-01-shSCYL2 cells (Fig. 3G, H). The xenografts with shSCYL2 had decreased levels of phosphorylated AKT at S473 and PTEN at STT compared to parental and shluc cells (Fig. 3I), likely because of downregulated SCYL2 expression. These findings indicate that SCYL2 expression might regulate tumor development through PTEN phosphorylation in vitro and in vivo.

### CHC associates with SCYL2 and PTEN

To elucidate the intracellular molecular mechanism of PTEN phosphorylation by SCYL2, we analyzed SCYL2-binding proteins using MS-based immunoprecipitation, and many types of SCYL2-binding proteins were identified (Supplementary Fig. 4A and Supplementary Table 2). To further investigate the functional associations of SCYL2-binding proteins, we performed Gene Ontology (GO) analysis using the R package [37, 38]. Biological Process (BP) analyses revealed correlations with protein folding. The Cell Component (CC) terms indicated that SCYL2-binding proteins were intensely involved in granules, vesicle lumen, and adhesion. From the Molecular Function (MF) terms, ATP-dependent or ATP-independent protein folding chaperones and the ability to bind phosphatases were associated with SCYL2 expression. Moreover, SCYL2 bound to PI3K and PTEN-related proteins (PI3K regulatory subunit 2, serine/threonine-protein kinase SMG1, and major vault protein) and downstream targets of AKT (ATP-citrate lyase [ACLY], forkhead box protein O1, and 40S ribosomal protein S3) (Supplementary Table 2), suggesting that SCYL2 may regulate the cytoplasmic vesicle formation, trafficking, and signal transduction pathways (Supplementary Fig. 4B).

**Fig. 4:**
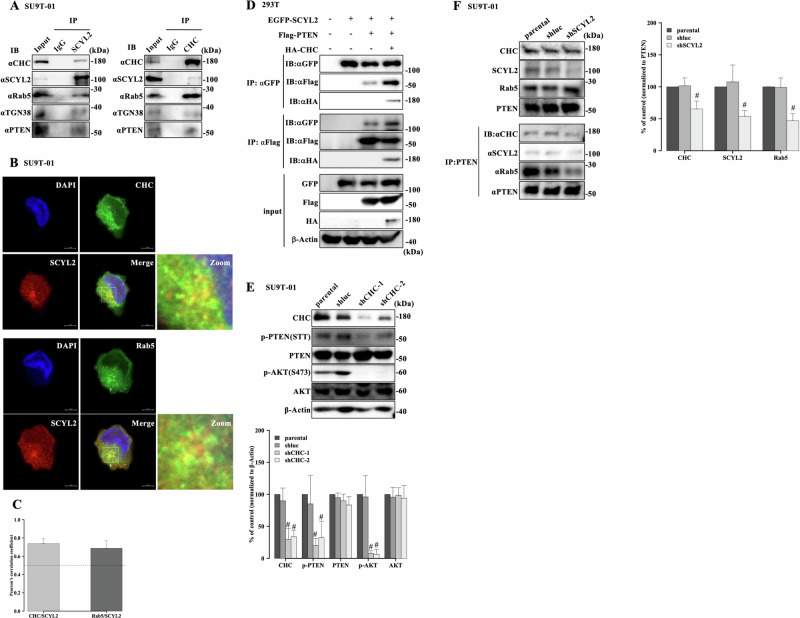
CHC associates with SCYL2 and PTEN. **A** Cell lysates from SU9T-01 were precipitated with anti-SCYL2 or -CHC antibodies, and the precipitated proteins were immunoblotted with the indicated antibodies. **B** CHC or Rab5 (Alexa Fluor-488, green) and SCYL2 (Alexa Fluor-555, red) were detected in SU9T-01 by immunofluorescent staining, and cell nuclei were stained with DAPI (blue). Scale bar, 5 µm. A region identified by the white box is further magnified to show the colocalization. **C** Quantification of colocalization of SCYL2 with CHC or Rab5 was carried out using Pearson’s correlation coefficient from 20 representative images. The minimal value for significant colocalization is 0.5 (dotted line). The mean and SD are shown (*n* = 20). **D** Cell lysates of 293T cells transfected with EGFP-SCYL2, Flag-PTEN, and HA-CHC were immunoprecipitated with anti-GFP or anti-Flag antibodies, and immunoprecipitates were detected by western blotting using the indicated antibodies. **E** Cell Lysate from SU9T-01 cells (parental, shluc, shCHC-1, and shCHC-2) was investigated using antibodies specific to each immunoblot. The results are representative of three independent experiments. Bar graphs show the quantification of relative band intensity normalized to β-actin. The mean and SD are shown (*n* = 3); #*p* < 0.05, versus parental. **F** Cell lysate form SU9T-01 cells (parental, shluc, and shSCYL2) was investigated using specific antibodies in immunoblots. PTEN immunoprecipitated from SU9T-01 cells were analyzed by immunoblotting with the indicated antibodies. The results are representative of three independent experiments. Bar graphs show the quantification of the relative band intensity normalized to immunoprecipitated PTEN. The mean and SD are shown (*n* = 3); #*p* < 0.05, versus parental.

SCYL2 reportedly binds to CHC, which is involved in tumorigenesis via clathrin-mediated endocytosis, and trans-Golgi trafficking. In addition, signaling molecules such as EGFP and PTEN are enriched in short-lived clathrin-coated pits, resulting in the regulation of signaling transduction pathways [28, 29, 39, 40]. Therefore, our MS results indicated that SCYL2 was associated with clathrin-binding proteins (cytoplasmic dynein 1 heavy chain 1, ACLY, and heat shock cognate 71 kDa protein) (Supplementary Table 2). Endogenous SCYL2 and CHC co-immunoprecipitated with each specific antibody and colocalized in the cytoplasm and perinuclear region of ATL cells. Furthermore, SCYL2 and CHC associated with PTEN, early endosome marker Rab5, and trans-Golgi networks marker TGN38, and Pearson’s correlation coefficient to estimate the degree of colocalization [41–43] confirmed significant colocalization of SCYL2 with CHC and Rab5 (Fig. 4A–C, and Supplementary Fig. 4C–E), suggesting that SCYL2 binds with PTEN-related proteins via clathrin-mediated endosome and trans-Golgi networks leading to the complex of signaling platforms.

Next, EGFP-SCYL2, Flag-PTEN, and/or HA-CHC were co-transfected into 293 T cells in different combinations, and each specific tagged antibody was used to detect the protein complex. PTEN was detected in the protein complexes in 293T cells transfected with SCYL2, and the protein complex between SCYL2 and PTEN was enhanced by transfection with CHC (Fig. 4D). Moreover, a tendency toward increased CHC expression was observed in primary ATL patient cells compared to PBMCs and CD4^+^ T cells (Supplementary Fig. 4F, G). Suppression of CHC expression by introducing shRNA against CHC in ATL cells reduced AKT phosphorylation at S473 and PTEN phosphorylation at the STT (Fig. 4E and Supplementary Fig. 4H).

To investigate whether the presence of CHC regulates PTEN (STT) phosphorylation, the kinase activity of Flag-SCYL2 and/or HA-CHC transiently expressed in 293T cells were purified by immunoprecipitation with each tagged antibody measured using an in vitro kinase assay with GST-PTEN as a substrate. The recombinant PTEN (STT) phosphorylation was increased by the immunoprecipitated CHC. Furthermore, the combination of immunoprecipitated SCYL2 and CHC markedly induced PTEN phosphorylation compared to SCYL2 or CHC alone (Supplementary Fig. 4I). Although CHC expression was unaffected by SCYL2 knockdown in ATL cell lines, the binding of PTEN to CHC and Rab5 was suppressed in shSCYL2 ATL cell lines compared with that in parental and shluc lines (Fig. 4F and Supplementary Fig. 4J, K). These results suggest that interactions between SCYL2 and CHC may lead to PTEN accumulation through the regulation of vesicle formation and trafficking as a signaling platform, followed by enhanced PTEN phosphorylation, which may become a therapeutic target for ATL cells.

### CCVs inhibitors suppress proliferation of ATL cells

**Fig. 5:**
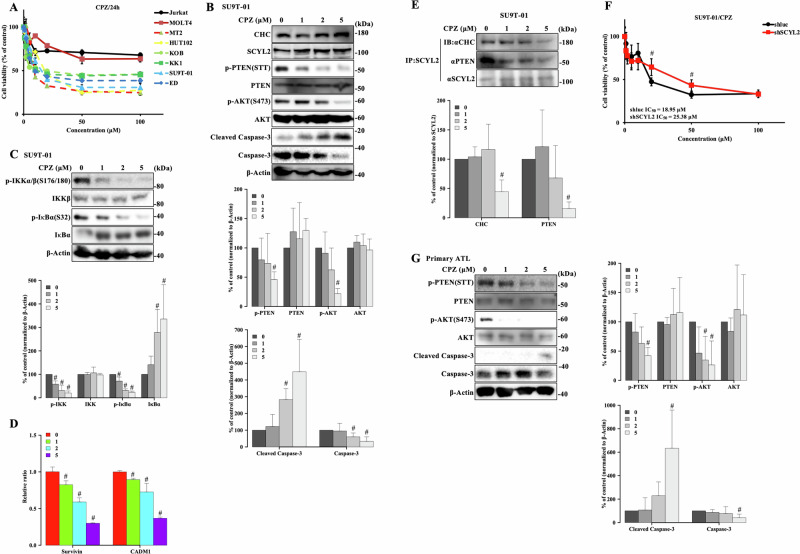
CCVs inhibitors suppress proliferation of ATL cells. **A** Cell viability and IC_50_ were determined using Cell Counting Kit-8 after treatment with 0–100 μM CPZ for 24 h in T-ALL and ATL-related cell lines. **B** SU9T-01 cells were treated with the indicated doses of CPZ for 24 h, and determined by immunoblot analysis. The results are representative of three independent experiments. Bar graphs show the quantification of the relative band intensity normalized to β-actin. The mean and SD are shown (*n* = 3); *p < 0.05 versus 0. **C** Cell lysate from SU9T-01 cells treated with the indicated doses of CPZ for 24 h was subjected to western blot analysis of NF-κB pathway. The results are representative of three independent experiments. Bar graphs show the quantification of relative band intensity normalized to β-actin. The mean and SD are shown (*n* = 3); #*p* < 0.05, versus 0. **D** Quantitative PCR analysis of NF-κB target genes in SU9T-01 cells treated with the indicated doses of CPZ for 24 h. The mean and SD are shown (*n* = 4); #*p* < 0.05, versus 0. **E** SCYL2 was immunoprecipitated from SU9T-01 cells treated with the indicated doses of CPZ for 24 h; it was then analyzed via immunoblotting using the indicated antibodies. Bar graphs show the quantification of the relative band intensity normalized to immunoprecipitated SCYL2. The mean and SD are shown (*n* = 3); #*p* < 0.05 versus 0. **F** Cell viability and IC_50_ were determined using Cell Counting Kit-8 after treatment with 0–100 μM CPZ for 24 h in Su9T-01 cells (shluc and shSCYL2). The mean and SD are shown (*n* = 4); #*p* < 0.05, versus shluc. **G** The primary ATL cells were treated with the indicated doses of CPZ for 24 h, and determined by immunoblot analysis. Bar graphs show the quantification of the relative band intensity normalized to β-actin. The mean and SD are shown (*n* = 3); *p < 0.05 versus 0.

CPZ has been reported to exert potential anti-cancer effects by inhibiting the assembly of CCVs [44, 45]. Therefore, we evaluated the efficacy of CPZ as a therapeutic agent in ATL cells for 24 h. CPZ treatment inhibited cell viability in ATL cell lines in a dose-dependent manner at 24 h, with IC_50_ values of 8.93–18.08 μM; however, the inhibitory effect in T-ALL cells did not reach IC_50_ (Fig. 5A and Supplementary Table 3). Therefore, there was a significant reduction in phosphorylated AKT and PTEN along with the induction of cell apoptosis detected by Cleaved Caspase-3 and the inhibition of NF-κB signaling pathway in a concentration-dependent manner, regardless of the unchanged SCYL2 and CHC protein expression levels (Fig. 5B–D and Supplementary Fig. 5A–D). Furthermore, the association between SCYL2 and CHC, PTEN was markedly reduced by CPZ treatment in the ATL cell lines (Fig. 5E and Supplementary Fig. 5E). CPZ-induced decrease in AKT phosphorylation was rescued by the PTEN inhibitor bpV(HOpic) in ATL cells (Supplementary Fig. 5F), suggesting that disruption of CCVs increases PTEN lipid phosphatase activity via dephosphorylation leading to the suppression of AKT phosphorylation in ATL cells.

The inhibition of SCYL2 expression in ATL cell lines indicated a lower sensitivity to CPZ treatment than the shluc control (Fig. 5F and Supplementary Fig. 5G). We further analyzed the anti-tumor effects of CPZ in primary ATL patient cells. Although ATL cells from one patient were resistant to CPZ, CPZ treatment effectively suppressed the proliferation of primary ATL cells, with IC_50_ values ranging from 41.88 to 84.29 μM at 24 h (Supplementary Table 3). In addition, AKT and PTEN phosphorylation was significantly decreased in primary ATL cells with the increase of cell apoptosis (Fig. 5G), suggesting that SCYL2 and CHC inhibition may be therapeutic targets for ATL cells.

## Discussion

The results of the present study indicate that SCYL2 regulates PTEN phosphorylation through association with CHC as a signaling platform. We also demonstrated in vitro and in vivo that SCYL2 contributes to tumorigenesis through PTEN phosphorylation at the STT cluster in the C-tail, followed by inactivation of PTEN phosphatase activity and activation of the PI3K/AKT signaling pathway.

Aberrant activation of multiple signal transduction pathways is observed in many types of tumors through an imbalance between protein phosphatase and kinase. PTEN localization, lipid phosphatase activity, and stability are regulated by phosphorylation of the C-tail, which contains a cluster of S/T residues: S370, S380, T382, T383, and S385 [7–10, 12, 13]. Increased expression of S380A, T382A, and T383A, but not S370A and S385A mutants, in ATL cells reduced cell viability and AKT phosphorylation. Therefore, we hypothesized that PTEN phosphorylation at STT (rather than at S370 and S385) may modulate PTEN phosphorylation activity and ATL development [4]. Although CK2, GSK3β, and liver kinase B1 reportedly function as major kinases for PTEN at all C-tail clusters, the specific kinase for PTEN at the STT remains unclear [18, 19, 32, 46, 47].

It has been reported that SCYL2 is a novel CCV-binding protein with an S/T kinase homology domain at the N-terminal, which act as a poly-L-lysine stimulated kinase; the C-terminal domain of SCYL2 associates with clathrin and AP2. However, although SCYL2 does not have any kinase activity, the N-terminal pseudokinase domain of SCYL2 mediates interactions with intracellular membranes to regulate clathrin function, the trans-Golgi network, endosomal compartments, and lysosomal degradation [20–24]. The N-terminal kinase domain of SCYL2 at positions 1–375 is sufficient for binding to the C-tail of PTEN, and SCYL2-binding proteins phosphorylate PTEN at STT. Furthermore, SCYL2 knockdown suppressed PTEN phosphorylation in ATL cells. CHC increased the binding of PTEN to SCYL2, and CHC knockdown suppressed cell viability and the PI3K/AKT signaling pathway through PTEN dephosphorylation. Membrane-associated receptors and molecules are concentrated by CCVs and intracellular trafficking, followed by cytoplasmic vesicles, early endosomes, late endosomes, and lysosomes for degradation or recycling of the endosome back into the membrane. CCVs with early endosomes contain receptors, signaling molecules (EGFR, PI3K, mast/stem cell growth factor receptor Kit, IL7 receptor, AKT, extracellular signal-regulated kinase, tyrosine-protein kinase JAK3, and signal transducer and activator of transcription 5) [39, 48–52], lipids (PIP2 and PIP3) [53, 54], and PTEN kinase candidates (CK2, proto-oncogene tyrosine-protein kinase c-Src, and GSK3β) [55–57], which are part of the signal transduction pathways, suggesting that CHC may recruit PTEN to cytoplasmic vesicles as a signaling platform in the presence of SCYL2 expression. This may be followed by tumor development and the activation of signaling pathways through PTEN phosphorylation (Supplementary Fig. 6A).

CPZ is an antipsychotic drug approved by the US Food and Drug Administration. It has been reported to have effective anti-cancer potential through the inhibition of CHC conformation and signaling pathways and through the induction of apoptosis and autophagy [44, 45]. Our results indicated that CPZ treatment significantly suppressed the cell viability rate and PI3K/AKT signaling pathway in ATL cells through PTEN dephosphorylation. In addition, CPZ was used in a Phase II clinical trial in patients with glioblastoma multiforme [58]. Suppression of SCYL2 expression or inhibition of CHC may therefore be a feasible and effective strategy for promoting the prevention and treatment of cancers (Supplementary Fig. 6B).

Our study had several limitations. First, we used a xenograft mouse model to investigate the in vivo effects of SCYL2 on tumorigenesis. Since *SCYL2* homozygous mice died shortly after birth, future studies are warranted to confirm the physiological and pathological functions of SCYL2 in T cell-specific animal models. Furthermore, it remains to be determined whether direct or additional factors involved in the SCYL2/CHC complex enhance the phosphorylation of AKT and PTEN.

In conclusion, we demonstrated that SCYL2 regulates PTEN phosphorylation at the STT via CCVs. The identification of new molecular mechanisms of the PI3K/AKT signaling pathway may lead to the development of new cancer treatments aimed at restoring PTEN function through its dephosphorylation. Therefore, inhibitors targeting SCYL2/CHC may be novel therapeutic agents for many malignancies, including ATL.

## Material and methods

### Reagents

CPZ was purchased from Sigma-Aldrich (St. Louis, MO, USA), and bpV(HOpic) was obtained from Selleck (Houston, TX, USA). ATP was purchased from TaKaRa Bio (Shiga, Japan). The cell proliferation/cell toxicity kit (Cell Counting Kit-8) was purchased from DOJINDO (Kumamoto, Japan). Most antibodies were purchased from companies listed in Supplementary Table 4. Rabbit polyclonal antisera against three SCYL2 peptides (TDNTKRNLTNGLNA, QLSQQKPNQWLNQFV, and TTMTNSSSASNDLKDLFG) were generated by Hokkaido System Science (Hokkaido, Japan).

The remaining Materials and Methods are presented in the Supporting Information.

## Supplementary information

Supplemental Material

## Data Availability

Publicly available data analyzed in this study were obtained from the Gene Expression Omnibus database (accession numbers GSE33615, GSE55851, and GSE43017). All other raw data generated in this study are available upon request from the corresponding author.
