# Supplementary material for: Clathrin-associated SCYL2 contributes to the activation of PI3K/AKT signaling and tumorigenesis through PTEN phosphorylation in adult T-cell leukemia/lymphoma
Source: Cancer Gene Ther. 2026 Feb 16;33(3):314–22. doi: 10.1038/s41417-026-01008-9 (PMC13109056; doi:10.1038/s41417-026-01008-9)
Supplement: Supplementary file 1 — Supplemental Material [file 41417_2026_1008_MOESM1_ESM.pdf]

Supplementary Information for

Clathrin-associated SCYL2 contributes to the activation of PI3K/AKT signaling and tumorigenesis through PTEN phosphorylation in adult T-cell leukemia/lymphoma

Tomonaga Ichikawa, Shunsuke Shimosaki, Shingo Nakahata, Akira Suekane, Issay Kitabayashi, Hidekatsu Iha, Kazuya Shimoda, Takashi Murakami, Kazuhiro Morishita.

Please send correspondence to Tomonaga Ichikawa, e-mail: to_ichi@saitama-med.ac.jp

Supplementary Material and Methods

Supplementary Tables (Supplementary Table 1-6)

Supplementary Figures (Supplementary Fig. 1-6)

Supplementary Material and Methods

Plasmids

Flag-tagged PTEN (Flag-PTEN), enhanced green fluorescent protein (EGFP)-tagged PTEN (EGFP-PTEN), PTEN-N (amino acids 1–185), and PTEN-C (amino acids 186–403) have been described previously[1]. Glutathione S-transferase (GST)-tagged PTEN (GST-PTEN) was generated using Flag-PTEN constructs as templates and subcloned into the pGEX-6P-1 expression vector (GE Healthcare, Uppsala, Sweden). EGFP-SCYL2 and serial deletion mutants (amino acids 1–697, 1–375, and 699–929) were kindly provided by Dr. E. Ungewickell (Hannover Medical School, Hannover, Germany)[2]. Flag-SCYL2 and serial deletion mutants were generated using EGFP-SCYL2 constructs as templates and subcloned into the p3xFlag-myc-CMV26 expression vector (Sigma-Aldrich). The shRNA vector with three different oligonucleotide DNA sequences against SCYL2 was cloned into the BamHI–EcoRI site of the RNAi-Ready-pSIREN-RetroQ-ZnGreen vector (shSCYL2) (Clontech, Mountain View, CA, USA). **Supplementary Table 5** lists the sense and antisense shRNA sequences. A plasmid containing shRNA against luciferase (shluc) was used as a control. Hemagglutinin (HA)-tagged CHC (HA-CHC) and pSUPER shRNA vector against control and CHC (shCHC) were kindly provided by Dr. M. Enari (National Cancer Center Research Institute, Tokyo, Japan). Transient transfections were performed using polyethylenimine hydrochloride (PEI-MAX; Polysciences, Warrington, PA, USA) and Amaxa Cell Line Nucleofector Kit V (LONZA, Basel, Switzerland) according to the manufacturer's protocol.

Generation of *Scyl2*-deficient mice

Scyl2-deficient mice were obtained from the Laboratory for Animal Resources and Genetic Engineering, RIKEN Center for Developmental Biology (Kobe, Japan). The resulting chimeras were bred with C57BL/6 mice, and heterozygous offspring were identified by PCR using the following primer pairs: wild-type (WT) allele, F1 (GCGTTTTCACTTTGTTGTGGTA) and R (TCCGACCTGTTTTGCACCAT); targeted allele, F2 (GCCAGAGGCCACTTGTGTAG), and R. Heterozygous mice were backcrossed to C57BL/6 and mated in the same generation to obtain homozygous mutants.

Xenograft tumor model

The injection sites of 6–8-week-old female NOD/Shi-scid, IL-2Rgnull (NOG) mice (CLEA Japan Inc., Tokyo, Japan) were shaved and cleaned with 70% ethanol. Next, the mice were randomly divided into three groups (n = 6), and subcutaneously injected into the anterior part of the right flank region with 1×10^7 SU9T-01 cells with parental, shluc, or shSCYL2 suspended in 100 µL PBS and mixed with an equal volume of Matrigel (BD Matrigel; BD Biosciences, Bedford, MA, USA). Tumor size was measured with calipers every 3 days, beginning 1 week after injection. The tumor volumes were calculated using the following formula: (length × width^2) × 0.5.

Cell lines

Jurkat and MOLT4 are HTLV-1-negative human T-cell acute lymphoblastic leukemia (T-ALL) cell lines. MT2 and HUT102 are human T cell lines transformed by HTLV-1 infection. KOB and KK1 are interleukin-2 (IL-2)-dependent ATL cell lines. SU9T-01 and ED are IL-2-independent ATL cell lines. Jurkat and MOLT4 cells were obtained from Fujisaki Cell Center (Hayashibara Biochemical Laboratories, Okayama, Japan). MT2 and HUT102 cells were kindly donated by Dr. H. Iha (Oita University, Oita, Japan). KOB and KK1 were kind gifts from Dr. Y. Yamada (Nagasaki University, Nagasaki, Japan). SU9T-01 was gifted by Dr. N. Arima (Kagoshima University, Kagoshima, Japan). The ED was kindly provided by Dr. M. Maeda (Kyoto University, Kyoto, Japan). Human embryonic kidney cell lines HEK293T (293T), HEK293GP (293GP), and cervical cancer cell line HeLa were obtained from the RIKEN Bioresource Center (Tsukuba, Japan). The osteosarcoma cell line U2OS (HTB-96) was purchased from American Type Culture Collection (Manassas, VA, USA). The prostate cancer cell line PC3 was a kind gift from Dr. T. Ochiya (National Cancer Center Research Institute, Tokyo, Japan). The procedure used to isolate mouse embryonic fibroblasts (MEF) from WT (+/+) and *Scyl2*-deficient (-/-) mice has been previously described[1]. IL-2-dependent ATL cells were maintained in RPMI 1640 medium (Nacalai Tesque, Kyoto, Japan) supplemented with 10% FBS and 10 ng/mL recombinant human IL-2 (Peprotech, Rocky Hill, NJ, USA) in a humidified atmosphere of 5% CO_2 at 37°C. HTLV-1-negative, HTLV-1-infected, and IL-2-independent ATL cell lines were maintained in the same medium, without IL-2. The other cells were cultured in Dulbecco's modified Eagle's medium (Nacalai Tesque) supplemented with 10% FBS. Mycoplasma contamination was routinely tested.

Patient samples

Blood samples were obtained with informed consent under the approval of the Institutional Review Board of the Faculty of Medicine, University of Miyazaki (Approval Code: 972, Approval Data: January 17, 2001), and the Ethical Committee of Saitama Medical University (Approval Code: 2023-013, Approval Data: July 11, 2023). Peripheral blood mononuclear cells (PBMCs) were isolated using Lymphoprep (Axis-Shield, Oslo, Norway). ATL cells were collected from patients at hospital admission before the start of chemotherapy. $CD4^+$ T lymphocytes were isolated from blood samples of healthy volunteers by negative selection using an AutoMACS with a $CD4^+$ T-cell isolation kit (Miltenyi Biotech, Bergisch Gladbach, Germany). The ATL cells were maintained in AIM-V medium (Thermo Fisher Scientific, Waltham, MA, USA) supplemented with 20% FBS, 10 mM 2-mercaptoethanol (Thermo Fisher Scientific), and 10 ng/mL recombinant human IL-2.

Stable gene knockdown in cancer cell lines

The shRNA vectors were co-transfected into 293GP cells using the envelope plasmid pVSV-G with PEI-MAX reagent, according to the manufacturer's instructions. After 6 h of transfection, the medium was changed and the cells were incubated for 48 h in Dulbecco's modified Eagle's medium supplemented with 10% FBS and 10 μM forskolin (Sigma-Aldrich). Retrovirus-containing supernatants were collected using polyethylene glycol (Fujifilm Wako Pure Chemical,

Osaka, Japan). Two days after retroviral infection of the cancer cell lines, ZnGreen-positive cells were sorted using a JSAN cell sorter (Bay Bioscience, Kobe, Japan).

Western blot

Cells were harvested for protein extraction by homogenization in NP-40 lysis buffer (50 mM Tris-HCl, pH 8.0, 150 mM NaCl, 5 mM EDTA, and 1% NP-40) supplemented with a proteinase inhibitor cocktail (Sigma-Aldrich) and phosphatase inhibitor tablet (PhosStop; Roche, Basel, Switzerland). The lysate was centrifuged at 15,000 × *g* (maximum) for 10 min at 4°C and the supernatant was collected. The supernatant protein concentration was measured using a bovine serum albumin (BSA) standard. Equal amounts of protein samples were boiled at 95°C for 10 min in 1× SDS sample buffer (62.5 mM Tris-HCl, pH 6.8, 2% SDS, 25% glycerol, 5% 2-mercaptoethanol, and 0.01% bromophenol blue), separated by SDS-PAGE, and transferred to a polyvinylidene difluoride membrane (Immobilon-P; Millipore, Bedford, MA, USA). The membranes were blocked in TBS (10 mM Tris-HCl, pH 7.4, 100 mM NaCl)-Tween (0.1%) (TBST) with 1% BSA or Blocking One (Nacalai Tesque) prior to incubation with primary antibodies (1:1000) in TBST-BSA or Can Get Signal Solution 1 (TOYOBO, Osaka, Japan) overnight at 4°C. After washing three times with TBST, the membranes were incubated with horseradish peroxidase-conjugated secondary antibodies diluted in TBST-BSA or Can Get Signal Solution 2 (TOYOBO) at room temperature for 1 h. Bands were detected using a Lumi-light Plus kit (Roche) and an LAS-3000 imager (Fujifilm, Tokyo, Japan). The band intensities were quantified using ImageJ software (National Institutes of Health, Bethesda, MD, USA).

Immunoprecipitation

Lysates were incubated with 1 μg of the indicated antibodies or normal IgG at 4°C overnight with constant rotation, followed by incubation with Protein G Sepharose 4 Fast Flow (GE Healthcare) for 2 h. Next, the immunoprecipitates were washed three times with PBS and the bound proteins were denatured in SDS sample buffer. Each sample was subjected to western blot.

cDNA synthesis and quantitative real-time PCR (qPCR)

Total RNA was isolated from the cells using NucleoSpin RNA Plus (TaKaRa Bio, Shiga, Japan). Next, cDNA was synthesized from 500 ng of RNA using an PrimeScript RT Master Mix (TaKaRa Bio). qPCR was performed using a StepOne Real-Time PCR System (Applied Biosystems, Carlsbad, CA, USA), LightCycler96 system (Roche), Brilliant III Ultra-Fast SYBR Green QPCR Master Mix (Agilent Technologies, Santa Clara, CA, USA), and THUNDERBIRD Next SYBR qPCR Mix (TOYOBO). The expression levels of the target genes were normalized to β-actin mRNA levels. **Supplementary table 6** lists all primer sequences.

Immunofluorescence

Cells were fixed with 4% paraformaldehyde for 10 min at room temperature, washed with TBS-0.1 M glycine, treated with 0.1% NP-40, and rewashed with TBS-0.1 M glycine. After blocking

with 1% BSA in TBS, the cells were incubated with primary antibodies (1:200) overnight at 4°C. The cells were then washed thrice with TBST and incubated with secondary antibodies (1:400) for 2 h at room temperature. The cells were washed three times with TBST, the cell nuclei were counterstained with 4',6-diamidino-2-phenylindole (DAPI) (Sigma-Aldrich), and the cells were then mounted onto glass slides using an antifade reagent (Thermo Fisher Scientific). Proteins were visualized using confocal laser-scanning microscope SP8 (Leica Microsystems, Wetzlar, Germany) and Zeiss LSM900 with Airyscan 2 (Zeiss, Oberkochen, Germany). Pearson's correlation coefficient is applied to measure colocalization within dual colour fluorescence images by ZEN 3.9 software.

Cell proliferation assay

Cells were seeded in 96-well plates at a density of 3×10^3 cells/well and incubated for the indicated time periods. Viable cells were counted using a methylthiazolyl tetrazolium assay with Cell Counting Kit-8. The 50% inhibitory concentration (IC_{50}) values were calculated using the following formula: $IC_{50} = 10^{\mathrm{LOG}(A/B) \times (50 - C)/(D - C) + \mathrm{LOG}(B)}$, where A is the concentration of the upper side of 50% absorbance, B is the concentration of the lower side of 50% absorbance, C is the rate of absorbance reduction at a concentration of B, and D is the rate of absorbance reduction at a concentration of A.

Mass spectrometry (MS)

MS was performed at the National Cancer Center Research Institute (Tokyo, Japan) and HTLV-1/ATL Facility, University of Miyazaki. Briefly, proteins binding to Flag-PTEN or SCYL2 were isolated by immunoprecipitation with antibodies against Flag or SCYL2, resolved by SDS-PAGE, and stained with Coomassie blue R250 (Bio-Rad, Hercules, CA, USA). The band corresponding to each protein was excised and subjected to in-gel digestion with MS-grade trypsin or Lys-C (Promega, Madison, WI, USA). The samples were analyzed using liquid chromatography/quadrupole ion trap/time-of-flight MS (LCMS-IT-TOF, Shimadzu, Kyoto, Japan), and all data were processed using the Mascot server (Matrix Science Inc., Boston, MA, USA).

Gene ontology (GO) analysis

All identified proteins were subjected to GO analysis using R package *clusterProfiler*[3,4]. The GO terms of all three categories—biological process (BP), cell component (CC), and molecular function (MF)—were significantly represented after correction (adjusted p-value < 0.0001).

***In vitro* kinase assay**

A modified *in vitro* kinase assay was performed, as previously described[5]. Briefly, extracts from 293T cells expressing Flag-tagged molecules and the BL21 strain of *Escherichia coli* expressing GST-tagged substrates were immunoprecipitated using anti-Flag M2 Affinity Gel (Sigma-Aldrich) and Glutathione Sepharose (GE Healthcare), respectively, and mixed. The

immunocomplexes were then washed three times with lysis buffer and twice with kinase buffer (25 mM Tris-HCl, pH 7.5, 5 mM β-glycerophosphate, 2 mM dithiothreitol, 10 mM $MgCl_2$, and 10 mM $MnCl_2$). Kinase reactions were performed for 60 min at 30°C in kinase buffer supplemented with 100 μM ATP. The reactions were stopped by the addition of SDS sample buffer, followed by boiling. The reaction products were subjected to immunoblotting.

In vitro **phosphatase assay**

PTEN phosphatase activity was assessed by measurement of inorganic phosphate liberated during *in vitro* PTEN phosphatase reactions converting PIP3 to PIP2. After immunoprecipitation, the beads were washed with phosphatase assay buffer containing 100 mM Tris-HCl (pH 8) and 10 mM Dithiothreitol (DTT) (Nacalai Tesque). The phosphatase reactions were performed in 50 μl of assay buffer and 200 μM water-soluble substrate phosphatidylinositol 3,4,5-trisphosphate diC8 (PIP3 diC8) (Echelon Bioscience, Salt Lake City, UT), and incubated for 40 min at 37°C. The release of phosphate from the substrate was measured in a colorimetric assay by using the Biomol Green Reagent (Enzo Life Science, Farmingdale, New York) in accordance with the instructions of the manufacturer. The absorbance at 630 nm was recorded in plate reader. A standard curve was performed in each assay, and the amount of free phosphate was calculated from the standard curve determined for inorganic phosphate.

Statistical analysis

No statistical methods were used to predetermine sample size. Sample size was defined according to our previous experience. Each experiment was independently performed at least three times. Results were not blinded for analysis. Data, bars, and markers in the figures represent mean ± SD. We used the two-tailed Student's *t*-test for comparisons within each parameter, and ANOVA and Dunnett's multiple comparisons test for multiple comparisons or to compare several different treatments with a single control. Statistical analyses were performed using Prism software 9 (GraphPad, San Diego, CA, USA) and R package. Differences were considered statistically significant at $p < 0.05$.

Supplementary Reference

1 Nakahata S, Ichikawa T, Maneesaay P, Saito Y, Nagai K, Tamura T *et al.* Loss of NDRG2 expression activates PI3K-AKT signalling via PTEN phosphorylation in ATLL and other cancers. *Nat Commun* 2014; **5**: 3393.

2 Dü M, Ungewickell EJ. Clathrin-dependent Association of CVAK104 with Endosomes and the Trans-Golgi Network □ D □ V. *Mol Biol Cell* 2006; **17**: 4513–4525.

3 Yu G, Wang LG, Han Y, He QY. ClusterProfiler: An R package for comparing biological themes among gene clusters. *OMICS* 2012; **16**: 284–287.

4 Wu T, Hu E, Xu S, Chen M, Guo P, Dai Z *et al.* clusterProfiler 4.0: A universal enrichment tool for interpreting omics data. *Innovation* 2021; **2**. doi:10.1016/j.xinn.2021.100141.

5 Mehenni H, Lin-Marq N, Buchet-Poyau K, Reymond A, Collart MA, Picard D *et al.* LKB1 interacts with and phosphorylates PTEN: A functional link between two proteins involved in cancer predisposing syndromes. *Hum Mol Genet* 2005; **14**: 2209–2219.

Supplementary Table 1. The list of proteins that co-immunoprecipitated with Flag-PTEN in ATL cells, identified by MS analysis.

Protein name	Gene name	Mr (Da)	Score	Matches	emPAI	Band ID
Kinesin-like protein KIF11	KIF11	119085	699	41	0.9	1
SCY1-like protein 2	SCYL2	103642	302	21	0.37	2
Protein arginine N-methyltransferase 5	PRMT5	72638	331	23	1.01	3
Heat shock cognate 71 kDa protein	HSP7C	70854	159	9	0.36	3
Heat shock 70 kDa protein 1	HSP71	70009	100	4	0.17	3
Heat shock-related 70 kDa protein 2	HSP72	69978	91	4	0.17	3
Heat shock 70 kDa protein 1L	HS70L	70331	69	4	0.05	3
Serum albumin precursor	ALBU	69321	64	5	0.05	3
78 kDa glucose-regulated protein precursor	GRP78	72288	50	6	0.05	3
Phosphatidylinositol-3,4,5-trisphosphate 3-phosphatase	PTEN	47136	835	54	2.95	4
Actin, cytoplasmic 1	ACTB	41710	437	22	2.33	5
Actin, alpha skeletal muscle	ACTS	42024	219	16	0.98	5
Actin, aortic smooth muscle	ACTA	41982	195	14	0.67	5
Methylosome protein 50	MEP50	36701	155	8	0.63	5
Elongation factor 1-alpha 1	EF1A1	50109	43	3	0.07	5

Supplementary Table 2. The list of proteins that co-immunoprecipitated with SCYL2 in ATL cells, identified by MS analysis.

Protein name	Gene name	Mr (Da)	Score	Matches	Sequences	BandI D	Enzyme
Cytoplasmic dynein 1 heavy chain 1	DYNC1H1	534809	145	27(7)	26(7)	1	Trypsin
Inositol 1,4,5-trisphosphate receptor type 1	ITPR1	317151	36	4(1)	4(1)	1	Trypsin
Lysosomal-trafficking regulator	LYST	434169	32	1(1)	1(1)	2	Lys-C
EH domain-binding protein 1-like protein 1	EHBP1L1	162554	44	5(1)	5(1)	2	Trypsin
Nuclear receptor corepressor 1	NCOR1	270957	37	1(1)	1(1)	2	Trypsin
Carboxypeptidase D	CPD	153919	37	2(1)	2(1)	2	Trypsin
2-oxoglutarate dehydrogenase complex component E1	OGDH	117059	188	7(4)	7(4)	3	Lys-C
Major vault protein	MVP	99551	162	6(4)	6(4)	3	Lys-C
Serine/threonine-protein kinase SMG1	SMG1	414347	33	3(1)	2(1)	3	Lys-C
Chromosome-associated kinesin KIF4A	KIF4A	141390	33	3(1)	2(1)	3	Lys-C
Junction plakoglobin	JUP	82434	32	1(1)	1(1)	3	Lys-C
Coiled-coil domain-containing protein 150	CCDC150	129536	30	2(1)	2(1)	3	Lys-C
Striatin	STRN	86535	346	19(8)	19(7)	3	Trypsin
SCY1-like protein 2	SCYL2	104327	306	15(10)	15(10)	3	Trypsin
Major vault protein	MVP	99551	129	8(3)	8(3)	3	Trypsin
Nucleolin	NCL	76625	112	9(2)	9(2)	3	Trypsin
Zinc finger CCHC domain-containing protein 8	ZCCHC8	79156	102	5(1)	5(1)	3	Trypsin
Splicing factor, proline- and glutamine-rich	SFPQ	76216	73	4(2)	4(2)	3	Trypsin
Endoplasmin	HSP90B1	92696	50	4(1)	4(1)	3	Trypsin
Heat shock 70 kDa protein 4	HSPA4	95127	45	3(1)	3(1)	3	Trypsin
ATP-citrate synthase	ACLY	121674	42	3(1)	3(1)	3	Trypsin
Striatin-4	STRN4	81287	41	6(1)	6(1)	3	Trypsin
Arf-GAP with SH3 domain, ANK repeat and PH domain-containing protein 1	ASAP1	126390	40	9(1)	9(1)	3	Trypsin

Pre-mRNA-processing factor 40 homolog A	PRPF40A	109022	38	1(1)	1(1)	3	Trypsin
Heterogeneous nuclear ribonucleoprotein U	HNRNPU	91269	35	8(1)	8(1)	3	Trypsin
Endoplasmic reticulum chaperone BiP	HSPA5	72402	199	14(7)	11(6)	4	Lys-C
Albumin	ALB	71317	124	12(4)	11(4)	4	Lys-C
Prelamin-A/C	LMNA	74380	54	4(1)	4(1)	4	Lys-C
MICOS complex subunit MIC60	IMMT	84026	46	4(2)	3(2)	4	Lys-C
Tripartite motif-containing protein 45	TRIM45	66256	36	1(1)	1(1)	4	Lys-C
WD repeat and coiled-coil-containing protein	WDCP	80283	35	1(1)	1(1)	4	Lys-C
tRNA dimethylallyltransferase	TRIT1	53035	35	1(1)	1(1)	4	Lys-C
Stress-70 protein, mitochondrial	HSPA9	73920	34	5(1)	5(1)	4	Lys-C
E3 ubiquitin-protein ligase TRIM68	TRIM68	57193	32	1(1)	1(1)	4	Lys-C
Phosphatidylinositol 3-kinase regulatory subunit beta	PIK3R2	81837	30	1(1)	1(1)	4	Lys-C
Stress-70 protein, mitochondrial	HSPA9	73920	497	24(14)	22(13)	4	Trypsin
Heat shock protein HSP 90-beta	HSP90AB1	83554	418	19(11)	18(11)	4	Trypsin
Endoplasmic reticulum chaperone BiP	HSPA5	72402	400	23(13)	21(13)	4	Trypsin
Heat shock cognate 71 kDa protein	HSPA8	71082	313	18(8)	16(8)	4	Trypsin
Heat shock protein HSP 90-alpha	HSP90AA1	85006	272	14(7)	18(11)	4	Trypsin
X-ray repair cross-complementing protein 5	XRCC5	83222	108	7(3)	7(3)	4	Trypsin
Prelamin-A/C	LMNA	74380	98	14(3)	12(3)	4	Trypsin
Heat shock 70 kDa protein 1-like	HSPA1L	70730	83	3(1)	3(1)	4	Trypsin
Heat shock protein 75 kDa, mitochondrial	TRAP1	80345	81	1(1)	1(1)	4	Trypsin
Striatin-3	STRN3	87554	69	2(1)	2(1)	4	Trypsin
Polyadenylate-binding protein 1	PABPC1	70854	64	6(2)	6(2)	4	Trypsin
Heat shock 70 kDa protein 6	HSPA6	71440	61	2(1)	2(1)	4	Trypsin
ATP-dependent RNA helicase DDX3X	DDX3X	73597	57	1(1)	1(1)	4	Trypsin
Polyadenylate-binding protein 1-like	PABPC1L	68976	44	3(1)	3(1)	4	Trypsin
Adenylate kinase 7	AK7	83120	44	2(1)	2(1)	4	Trypsin

RNA-binding protein EWS	EWSR1	68721	44	2(1)	2(1)	4	Trypsin
WD repeat and coiled-coil-containing protein	WDCP	80283	42	2(1)	1(1)	4	Trypsin
Far upstream element-binding protein 2	KHSRP	73355	42	4(1)	4(1)	4	Trypsin
Cilia- and flagella-associated protein 52	CFAP52	69281	38	1(1)	1(1)	4	Trypsin
Albumin	ALB	71317	37	6(1)	6(1)	4	Trypsin
Sodium-dependent phosphate transport protein 3	SLC17A2	47873	37	1(1)	1(1)	4	Trypsin
Forkhead box protein O1	FOXO1	70017	36	1(1)	1(1)	4	Trypsin
Actin, cytoplasmic 1	ACTB	42052	86	3(3)	3(3)	5	Lys-C
E3 ubiquitin-protein ligase RNF149	RNF149	43707	31	1(1)	1(1)	5	Lys-C
Actin, cytoplasmic 1	ACTB	42052	163	7(5)	7(5)	5	Trypsin
Actin, aortic smooth muscle	ACTA2	42381	131	6(3)	6(3)	5	Trypsin
Beta-actin-like protein 2	ACTBL2	42318	92	2(2)	2(2)	5	Trypsin
HLA class I histocompatibility antigen, B alpha chain	HLA-B	40777	34	1(1)	1(1)	5	Trypsin
Glyceraldehyde-3-phosphate dehydrogenase	GAPDH	36201	207	5(5)	4(4)	6	Trypsin
Elongation factor 1-delta	EEF1D	31217	43	1(1)	1(1)	6	Trypsin
Coiled-coil domain-containing protein 25	CCDC25	24634	36	2(1)	2(1)	6	Trypsin
Dermcidin	DCD	11391	37	1(1)	1(1)	7	Lys-C
Histone H1.4	H1-4	21852	35	1(1)	1(1)	7	Lys-C
MICOS complex subunit MIC19	CHCHD3	26421	30	1(1)	1(1)	7	Lys-C
60S ribosomal protein L18	RPL18	21735	51	1(1)	1(1)	7	Trypsin
40S ribosomal protein S3	RPS3	26842	49	4(1)	4(1)	7	Trypsin
Peroxiredoxin-2	PRDX2	22049	40	1(1)	1(1)	7	Trypsin
Peroxiredoxin-1	PRDX1	22324	37	3(1)	3(1)	7	Trypsin
Phospholipid phosphatase-related protein type 4	PLPPR4	83957	34	1(1)	1(1)	7	Trypsin

Supplementary Table 3. Inhibitory effect of CPZ on cell proliferation of ATL cell lines and primary ATL patient cells. The numbers represent IC_{50} (μM).

Cell origine	IC_{50} (μM)
Jurkat	>100
MOLT4	>100
MT2	8.93
HUT102	17.47
KOB	18.08
KK1	11.09
SU9T-01	14.34
ED	9.59
ATL#1	41.88
ATL#2	84.29
ATL#3	53.63
ATL#4	>100

Supplementary Table 4. The list of the antibodies used in this manuscript.

Antibody	Manufacturer	Catalog no	Type
AKT	Cell Signaling	#9272	Rabbit polyclonal
p-AKT(Ser473)	Cell Signaling	#9271	Rabbit polyclonal
PTEN(138G6)	Cell Signaling	#9559	Rabbit monoclonal
PTEN(26H9)	Cell Signaling	#9556	Mouse monoclonal
p-PTEN(Ser380/Thr382/383)	Cell Signaling	#9554	Rabbit polyclonal
Cleaved Caspase-3(Asp175)(5A1E)	Cell Signaling	#9664	Rabbit monoclonal
Caspase-3(D3R6Y)	Cell Signaling	#14220	Rabbit monoclonal
p-IKKα/β(Ser176/180)(16A6)	Cell Signaling	#2697	Rabbit monoclonal
IKKβ(D30C6)	Cell Signaling	#8943	Rabbit monoclonal
IκBα(L35A5)	Cell Signaling	#4814	Mouse monoclonal
p-IκBα(Ser32)(14D4)	Cell Signaling	#2859	Rabbit monoclonal
GFP(D5.1)	Cell Signaling	#2956	Rabbit monoclonal
DYKDDDK Tag(D6W5B)(Flag)	Cell Signaling	#14793	Rabbit monoclonal
Rab5(D-11)	SANTA CRUZ	sc-1637	Mouse monoclonal
TGN38(B-6)	SANTA CRUZ	sc-166594	Mouse monoclonal
GFP(B-2)	SANTA CRUZ	sc-9996	Mouse monoclonal
Flag(M2)	Sigma-Aldrich	F3165	Mouse monoclonal
β-actin(AC-74)	Sigma-Aldrich	A5316	Mouse monoclonal
HA(3F10)	Roche	11867423001	Rat monoclonal
SCYL2	Proteintech	12325-1-AP	Rabbit polyclonal
CHC(23)	BD	610500	Mouse monoclonal
p-PTEN(Ser370)	Millipore	07-889	Rabbit polyclonal
p-PTEN(Ser385)	Thermo Fisher	44-1064G	Rabbit polyclonal
Alexa Fluor-488 donkey anti-mouse	Thermo Fisher	A21202	
Alexa Fluor-555 donkey anti-rabbit	Thermo Fisher	A31572	
Polyclonal Rabbit anti-Mouse IgG/HPR	Dako	P0260	
Polyclonal Swine anti-Rabbit IgG/HPR	Dako	P0399	
Polyclonal Rabbit anti-Rat IgG/HPR	Dako	P0450	

Supplementary Table 5. The primer sequences to construct expression vectors with shRNA sequences.

Name		Sequence (5' to 3')
shSCYL2-1	sense	GCCCGTTAATACAAACCAG
	antisense	CTGGTTTGTATTAACGGGC
shSCYL2-2	sense	TGGGAAATCCTGTCACTAG
	anti-ense	CTAGTGACAGGATTTCCCA
shSCYL2-3	sense	ACCTGAGGAAGTTCGTGAA
	antisense	TTCACGAACTTCCTCAGGT

Supplementary Table 6. Primer list for real-time qPCR.

Gene		Sequence (5' to 3')
SCYL2	F	ACAAAGCAGGAAGTGGCAGT
	R	GAAGTCGAGGGTGTCGAAGC
CHC	F	GGCCCAGATTCTGCCAATTCGTTT
	R	TGATGGCGCTGTCTGCTGAAATTG
Bcl-2	F	CGGTGGGGTCATGTGTGTG
	R	CGGTTCAGGTACTCAGTCATCC
Survivin	F	GCCCAGTGTTTCTTCTGCTT
	R	GACAGAAAGGAAAGCGCAAC
CCND3	F	AGATCAAGCCGCACATGCGGAA
	R	ACGCAAGACAGGTAGCGATCCA
IFNγ	F	GAGTGTGGAGACCATCAAGGAAG
	R	TGCTTTGCGTTGGACATTCAAGTC
CADM1	F	GTCCCACCACGTAATCTGATG
	R	CCACCTCCGATTTGCCTTTA
β-actin	F	GACAGGATGCAGAAGGAGAT
	R	TGATCCACATCTGCTGGAAGGT

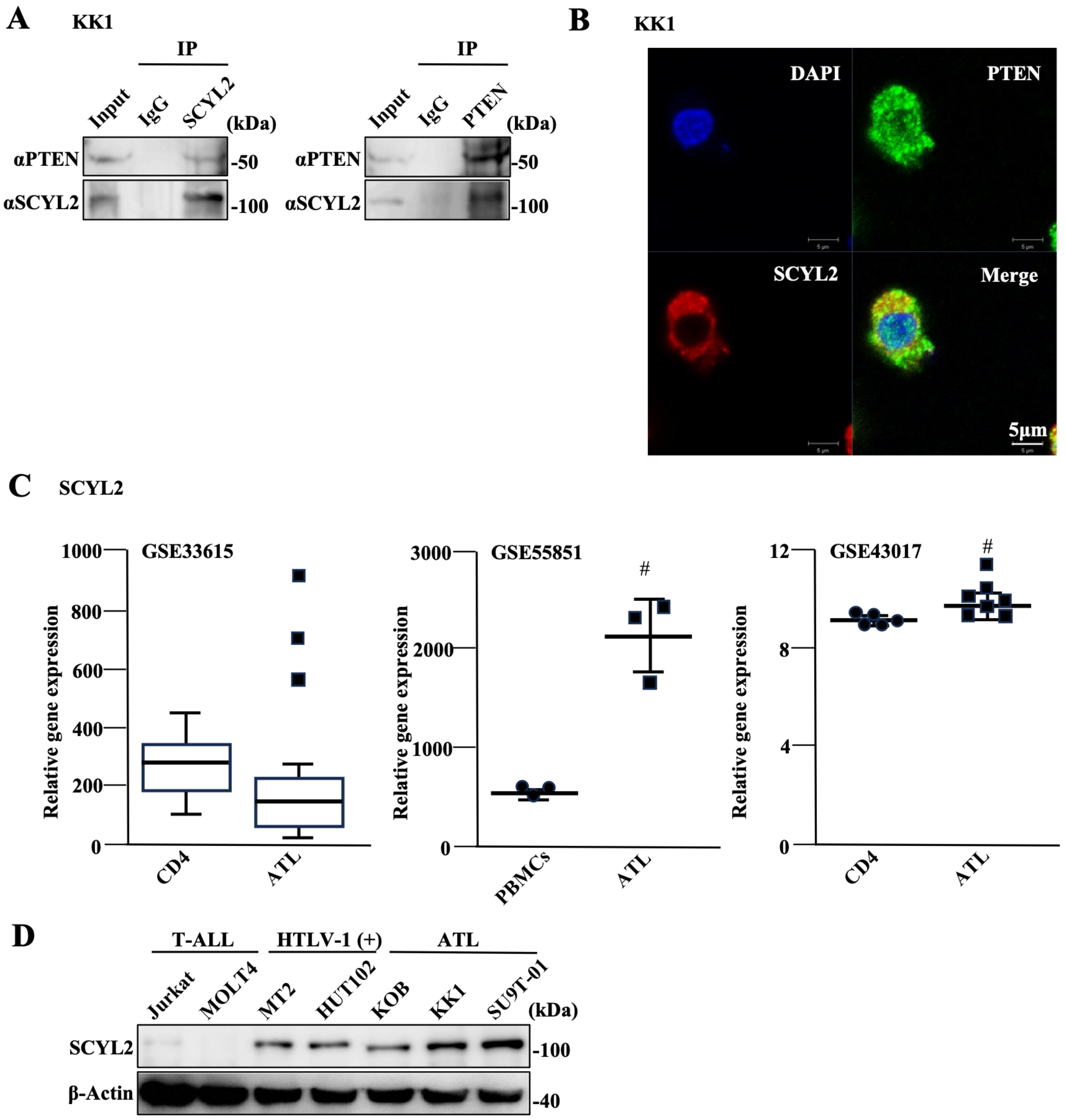
A
KK1
IP
Input
IgG
SCYL2
(kDa)
αPTEN
-50
αSCYL2
-100
IP
Input
IgG
PTEN
(kDa)
αPTEN
-50
αSCYL2
-100
B
KK1
DAPI
PTEN
SCYL2
Merge
5μm
C
SCYL2
GSE33615
Relative gene expression
1000
800
600
400
200
0
CD4
ATL
GSE55851
#
3000
2000
1000
0
PBMCs
ATL
GSE43017
#
12
8
4
0
CD4
ATL
D
T-ALL
HTLV-1 (+)
ATL
Jurkat
MOLT4
MT2
HUT102
KOB
KK1
SU9T-01
(kDa)
SCYL2
-100
β-Actin
-40

Supplementary Fig. 1.

A, Cell lysates from ATL cell line KK1 were precipitated using anti-SCYL2 or anti-PTEN antibody, and the precipitated proteins were immunoblotted with each specific antibody.

B, PTEN and SCYL2 were detected in KK1 by immunofluorescent staining using anti-PTEN antibody with Alexa Fluor-488-conjugated anti-mouse IgG antibody (green) or anti-SCYL2 with Alexa Fluor-555-conjugated anti-rabbit IgG antibody (red); the cell nuclei were stained with DAPI (blue). Scale bar, 5 μm.

C, Gene expression data obtained from Gene Expression Omnibus at NCBI. Data were presented as box and whisker plots showing the median, the box extends from 25% to 75% (Q1-Q3), and the whiskers connect the remaining lowest and highest 25%. Box and whisker plot of relative gene expression levels of *SCYL2* in CD4 (n = 20) and acute ATL patients (n = 26) (GSE33615). Dot plot of relative gene expression levels of SCYL2 in PBMCs (n = 3) and CDAM1(+)/CD7(-) ATL patients (n = 3) (GSE55851). Data were showed as the mean and SD; #$p < 0.05$ versus PBMCs. Dot plot of relative gene expression levels of SCYL2 in CD4 (n = 5) and acute ATL patients (n = 7) (GSE43017). Data were showed as the mean and SD; #$p < 0.05$ versus CD4.

D, Western blot analysis with the indicated antibodies was performed in the non-HTLV-1 T-ALL (Jurkat and MOLT4), HTLV-1-infected (MT2 and HUT102), and ATL (KOB, KK1, and SU9T-01) cell lines.

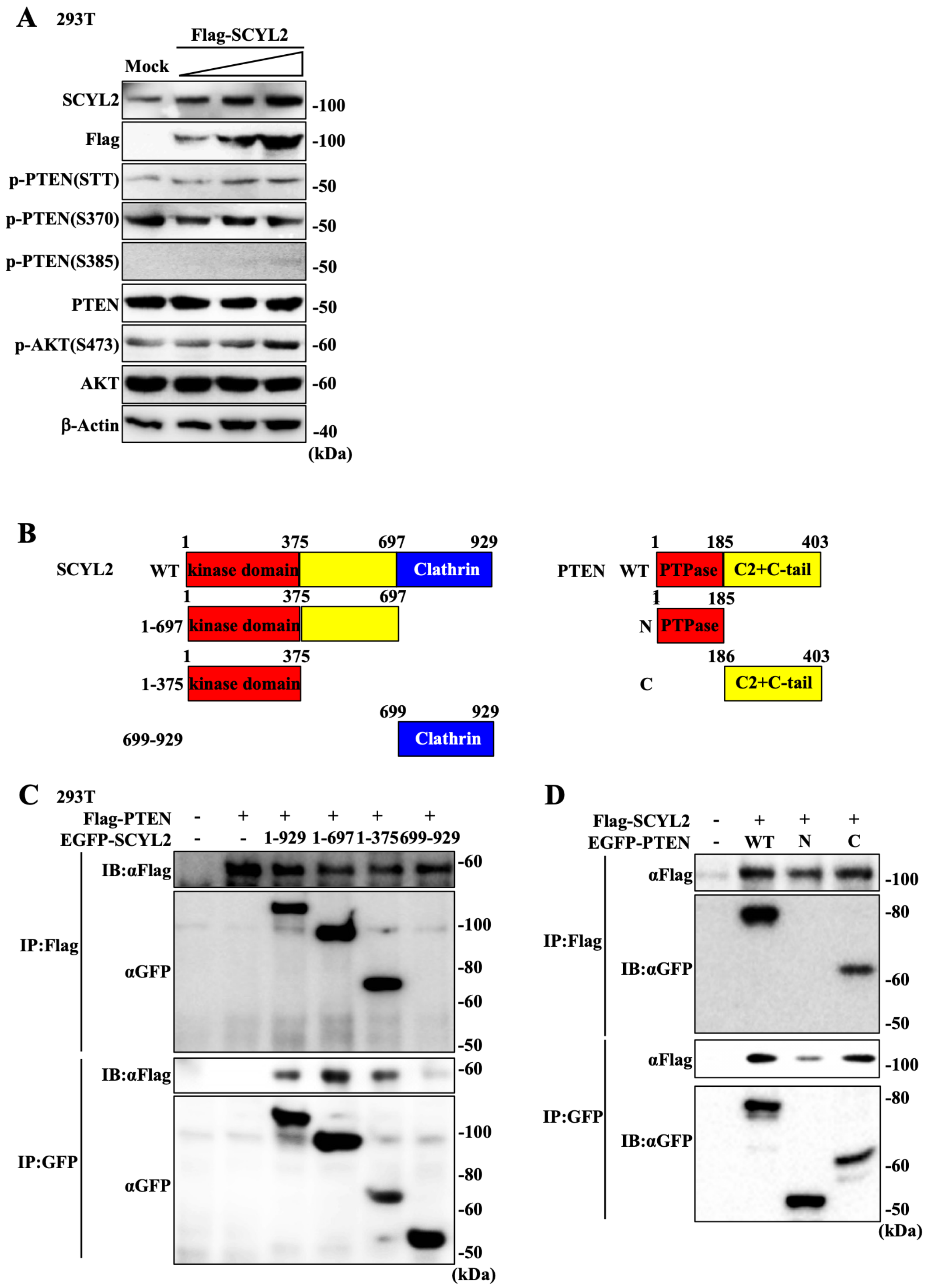
A
293T
Flag-SCYL2
Mock
SCYL2
Flag
p-PTEN(STT)
p-PTEN(S370)
p-PTEN(S385)
PTEN
p-AKT(S473)
AKT
β-Actin
-100
-100
-50
-50
-50
-50
-60
-60
-40
(kDa)
B
SCYL2
WT
1
375
697
929
kinase domain
Clathrin
1-697
1-375
699-929
699
PTEN
WT
1
185
403
PTPase
C2+C-tail
N
C
186
C
293T
Flag-PTEN
EGFP-SCYL2
1-929
1-697
1-375
699-929
IP:Flag
IP:GFP
IB:αFlag
αGFP
-60
-100
-80
-50
(kDa)
D
Flag-SCYL2
EGFP-PTEN
WT
N
C
αFlag
IB:αGFP
(kDa)

Supplementary Fig. 2.

A, 293T cells were transfected with increasing amounts of Flag-SCYL2 and subjected to western blot analysis with the indicated antibodies.

B, Schema of the structure of SCYL2 or PTEN and each deletion mutant. 1–375, kinase domain; 699–929, putative clathrin interaction motif; N, PTPase domain; C, C2+C-tail.

C, 293T cells were co-transfected with EGFP-SCYL2 mutant and Flag-PTEN, and immunoprecipitates were immunoblotted with anti-GFP or anti-Flag antibodies to detect SCYL2 and PTEN.

D, 293T cells were co-transfected with EGFP-PTEN mutant and Flag-SCYL2, and immunoprecipitates were immunoblotted with anti-GFP or anti-Flag antibodies to detect PTEN and SCYL2.

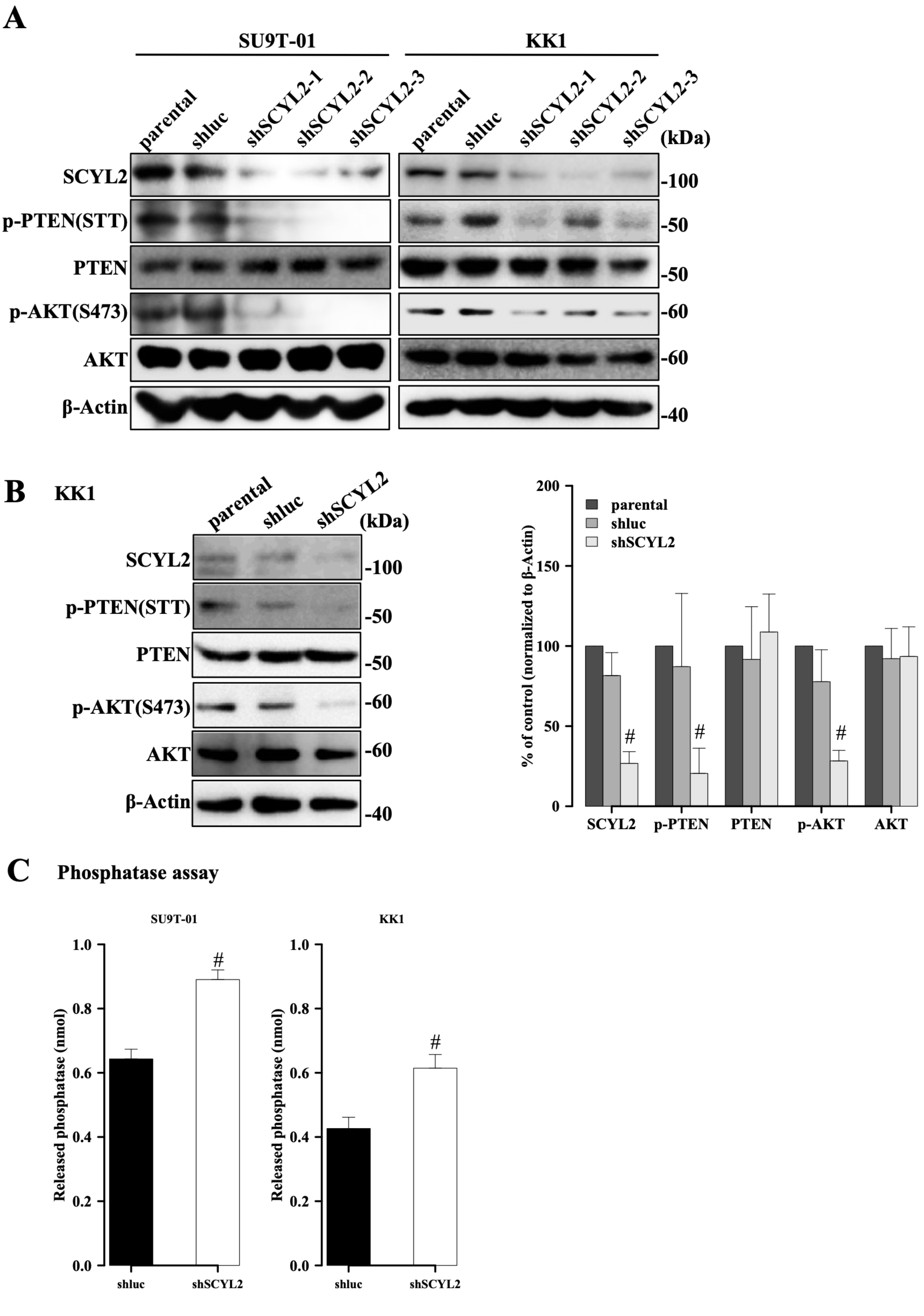
A
SU9T-01
KK1
parental
shluc
shSCYL2-1
shSCYL2-2
shSCYL2-3
(kDa)
SCYL2
p-PTEN(STT)
PTEN
p-AKT(S473)
AKT
β-Actin
-100
-50
-50
-60
-60
-40
B
KK1
parental
shluc
shSCYL2
(kDa)
SCYL2
p-PTEN(STT)
PTEN
p-AKT(S473)
AKT
β-Actin
-100
-50
-50
-60
-60
-40
% of control (normalized to β-Actin)
200
150
100
50
0
parental
shluc
shSCYL2
#
#
#
SCYL2
p-PTEN
PTEN
p-AKT
AKT
C
Phosphatase assay
SU9T-01
KK1
Released phosphatase (nmol)
1.0
0.8
0.6
0.4
0.2
0.0
#
#
shluc
shSCYL2
shluc
shSCYL2

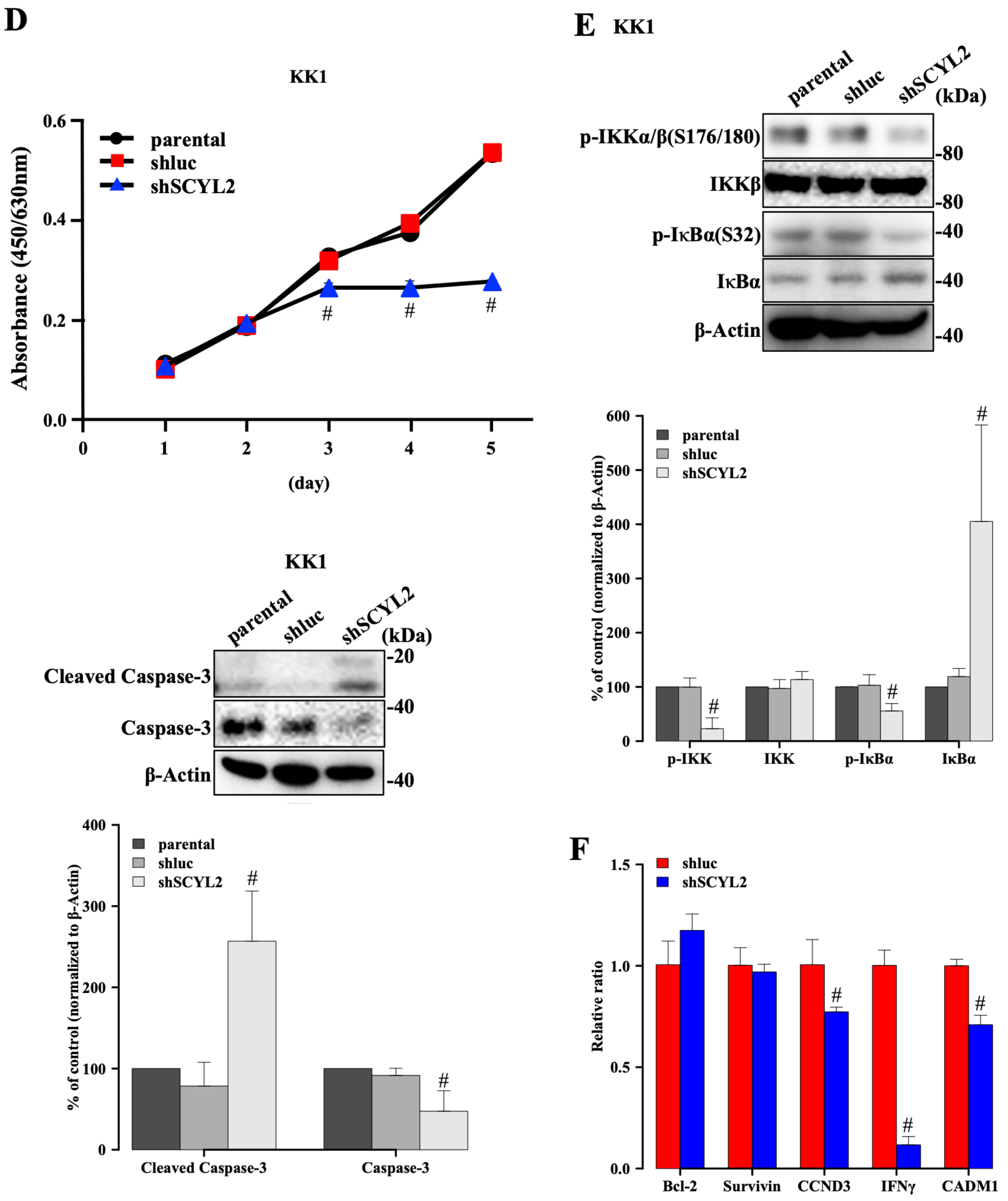
D
KK1
Absorbance (450/630nm)
parental
shluc
shSCYL2
0.0
0.2
0.4
0.6
0
1
2
3
4
5
(day)
#
#
#
KK1
parental
shluc
shSCYL2
(kDa)
Cleaved Caspase-3
Caspase-3
β-Actin
-20
-40
-40
% of control (normalized to β-Actin)
400
300
200
100
0
parental
shluc
shSCYL2
Cleaved Caspase-3
Caspase-3
E
KK1
parental
shluc
shSCYL2
(kDa)
p-IKKα/β(S176/180)
IKKβ
p-IκBα(S32)
IκBα
β-Actin
-80
-80
-40
-40
-40
% of control (normalized to β-Actin)
600
500
400
300
200
100
0
parental
shluc
shSCYL2
p-IKK
IKK
p-IκBα
IκBα
F
Relative ratio
1.5
1.0
0.5
0.0
shluc
shSCYL2
Bcl-2
Survivin
CCND3
IFNγ
CADM1

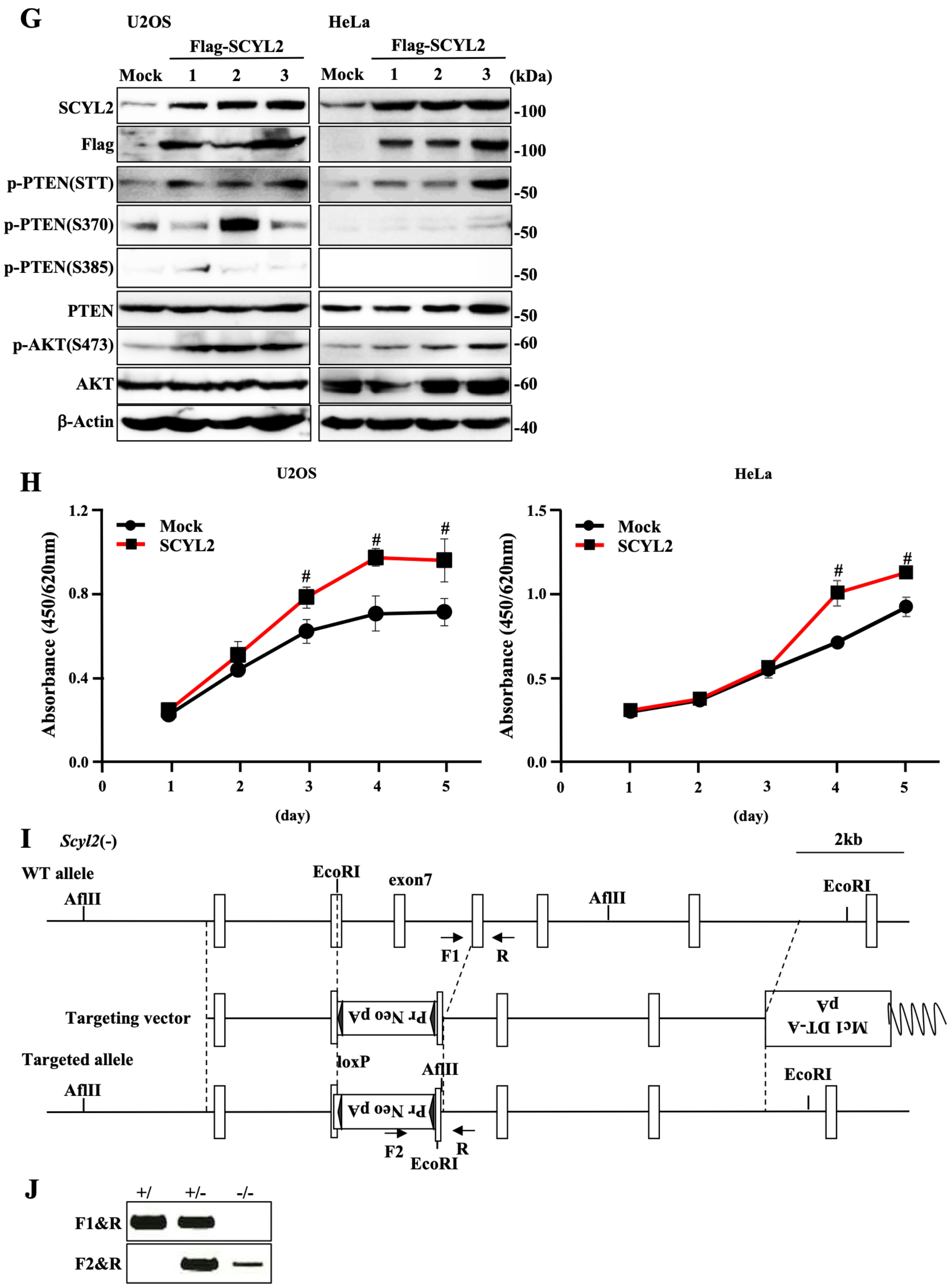
G
U2OS
HeLa
Flag-SCYL2
Flag-SCYL2
Mock 1 2 3
Mock 1 2 3 (kDa)
SCYL2 -100
Flag -100
p-PTEN(STT) -50
p-PTEN(S370) -50
p-PTEN(S385) -50
PTEN -50
p-AKT(S473) -60
AKT -60
β-Actin -40
H
U2OS
HeLa
Mock
SCYL2
Absorbance (450/620nm)
(day)
I
Scyl2(-)
2kb
WT allele
Targeting vector
Targeted allele
AflII
EcoRI
exon7
F1
F2
R
loxP
Pr Neo pA
J
+/ +/- -/-
F1&R
F2&R

Supplementary Fig. 3.

A, Expression of SCYL2 and PI3K/AKT signaling pathway in SU9T-01 and KK1 cells (parental, shluc, shSCYL2-1, -2, and -3) was investigated using each specific antibody in immunoblots.

B, Cell lysate from KK1 cells (parental, shluc, and shSCYL2) was investigated using antibodies specific to each immunoblot. The results are representative of three independent experiments. Bar graphs show the quantification of relative band intensity normalized to β-actin. The mean and SD are shown ($n = 3$); #$p < 0.05$, versus parental.

C, After PTEN immunoprecipitation from ATL cells, beads were incubated with PIP3 diC8, and phosphatase release was measured. The amount of released phosphatase was quantitated using the malachite green assay. The mean and SD are shown ($n = 4$); #$p < 0.05$, versus shluc.

D, Cell growth curves of KK1 cells for 5 days. The mean and SD are shown ($n = 4$); #$p < 0.05$, versus parental. The cell apoptosis (Cleaved Caspase-3 and Caspase-3) was investigated using each specific antibody in immunoblots. The results are representative of three independent experiments. Bar graphs show the quantification of relative band intensity normalized to β-actin. The mean and SD are shown ($n = 3$); #$p < 0.05$, versus parental.

E, Cell lysate from KK1 cells (parental, shluc, and shSCYL2) was subjected to western blot analysis of NF-κB pathway. The results are representative of three independent experiments. Bar graphs show the quantification of relative band intensity normalized to β-actin. The mean and SD are shown ($n = 3$); #$p < 0.05$, versus parental.

F, Quantitative PCR analysis of NF-κB target genes in KK1 cells (shluc and shSCYL2). The mean and SD are shown ($n = 4$); #$p < 0.05$, versus shluc.

G, Expression of SCYL2, p-PTEN (STT), p-PTEN (S370), p-PTEN (S385), PTEN, p-AKT, and AKT in solid cancer cell lines U2OS and HeLa (Mock, Flag-SCYL2-1, -2, and -3) was investigated using each specific antibody in immunoblots.

H, Cell growth curves of U2OS and HeLa cells (Mock and SCYL2) for 5 days. The mean and SD are shown (n = 4); #p < 0.05 versus Mock.

I, Schematic of the SCYL2 gene targeting strategy. Structure of the wild-type (WT) SCYL2 allele (top), the targeting vector (middle) and the predicted targeted allele (bottom). The Pr Neo pA cassette was inserted into exon 6 to 8 of the SCYL2 gene. PCR primers (small arrows, F1, F2 and R) were used to screen for homologous recombination.

J, PCR amplification of the WT (F1&R) and the targeted (F2&R) alleles resulted in products of 386 bp and 411 bp, respectively.

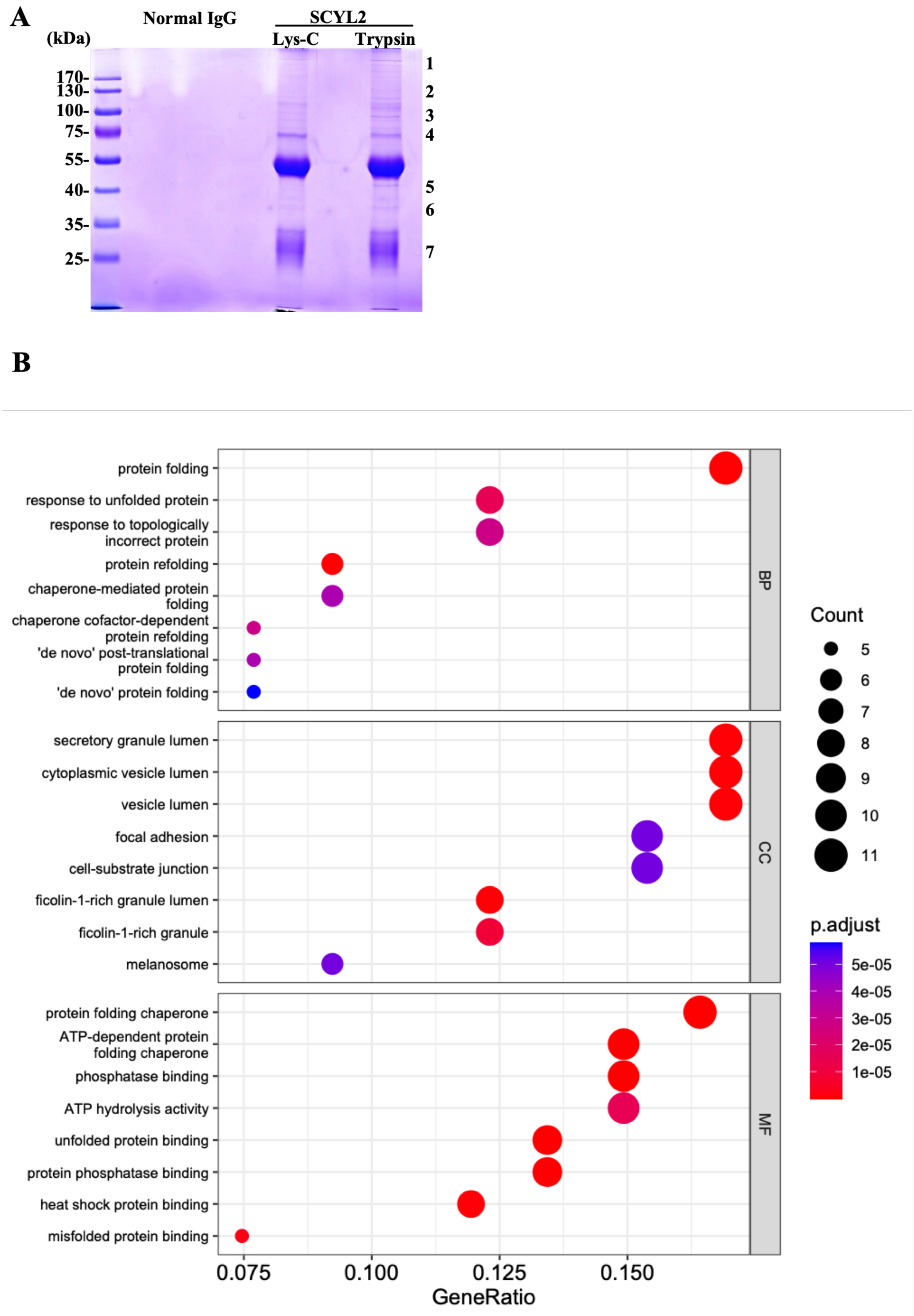
A
Normal IgG
SCYL2
(kDa)
Lys-C
Trypsin
170-
130-
100-
75-
55-
40-
35-
25-
1
2
3
4
5
6
7
B
protein folding
response to unfolded protein
response to topologically incorrect protein
protein refolding
chaperone-mediated protein folding
chaperone cofactor-dependent protein refolding
'de novo' post-translational protein folding
'de novo' protein folding
secretory granule lumen
cytoplasmic vesicle lumen
vesicle lumen
focal adhesion
cell-substrate junction
ficolin-1-rich granule lumen
ficolin-1-rich granule
melanosome
protein folding chaperone
ATP-dependent protein folding chaperone
phosphatase binding
ATP hydrolysis activity
unfolded protein binding
protein phosphatase binding
heat shock protein binding
misfolded protein binding
BP
CC
MF
0.075
0.100
0.125
0.150
GeneRatio
Count
5
6
7
8
9
10
11
p.adjust
5e-05
4e-05
3e-05
2e-05
1e-05

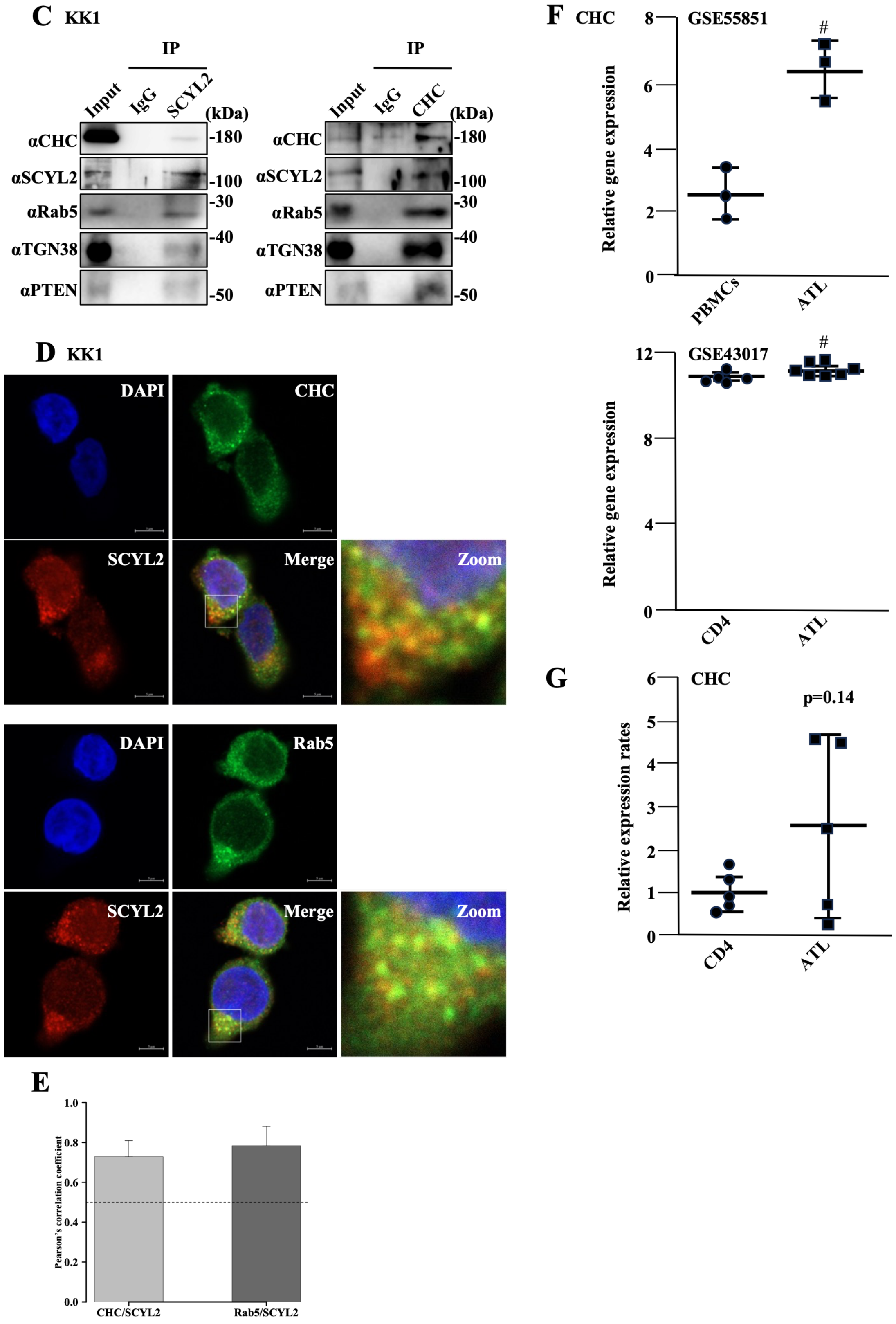
C KK1
IP
Input
IgG
SCYL2
(kDa)
αCHC
αSCYL2
αRab5
αTGN38
αPTEN
-180
-100
-30
-40
-50
IP
Input
IgG
CHC
(kDa)
αCHC
αSCYL2
αRab5
αTGN38
αPTEN
-180
-100
-30
-40
-50
D KK1
DAPI
CHC
SCYL2
Merge
Zoom
DAPI
Rab5
SCYL2
Merge
Zoom
E
Pearson's correlation coefficient
1.0
0.8
0.6
0.4
0.2
0.0
CHC/SCYL2
Rab5/SCYL2
F CHC
GSE55851
#
Relative gene expression
8
6
4
2
0
PBMCs
ATL
GSE43017
#
Relative gene expression
12
8
4
0
CD4
ATL
G
CHC
p=0.14
Relative expression rates
6
5
4
3
2
1
0
CD4
ATL

H

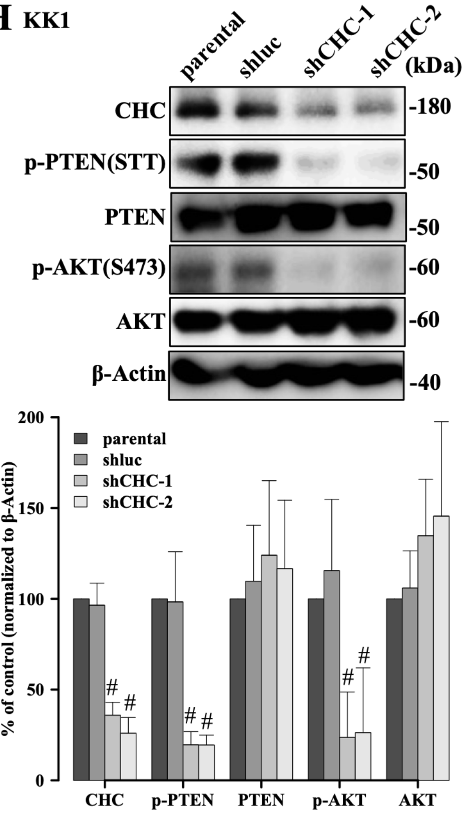
KK1
parental
shluc
shCHC-1
shCHC-2
(kDa)
CHC
-180
p-PTEN(STT)
-50
PTEN
-50
p-AKT(S473)
-60
AKT
-60
β-Actin
-40
% of control (normalized to β-Actin)
200
150
100
50
0
parental
shluc
shCHC-1
shCHC-2
#
CHC
p-PTEN
PTEN
p-AKT
AKT

I

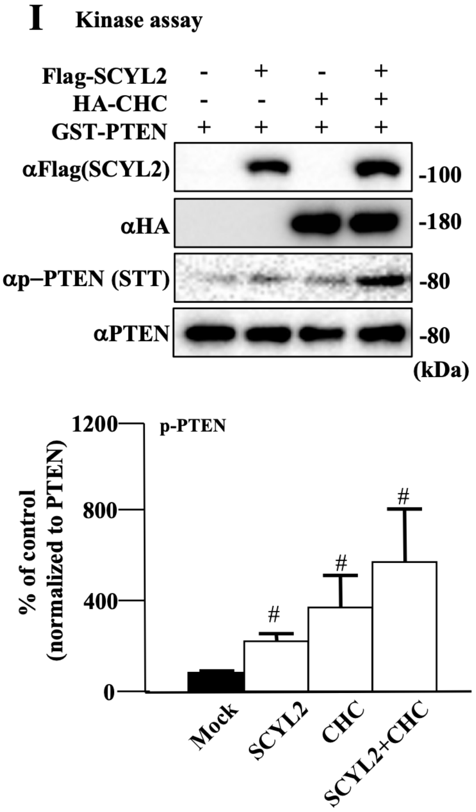
Kinase assay
Flag-SCYL2
HA-CHC
GST-PTEN
αFlag(SCYL2)
-100
αHA
-180
αp–PTEN (STT)
-80
αPTEN
-80
(kDa)
p-PTEN
% of control (normalized to PTEN)
1200
800
400
0
#
Mock
SCYL2
CHC
SCYL2+CHC

J

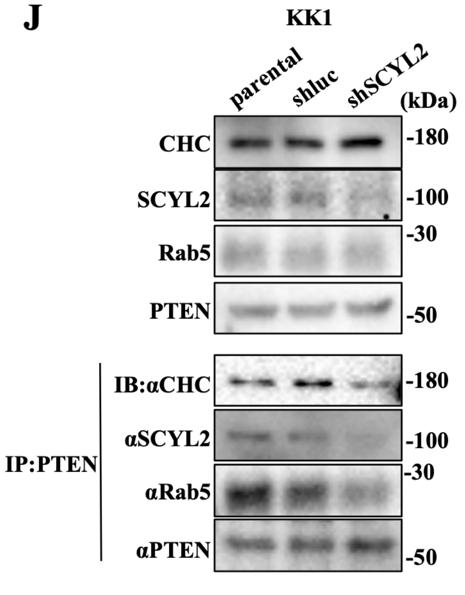
KK1
parental
shluc
shSCYL2
(kDa)
CHC
-180
SCYL2
-100
Rab5
-30
PTEN
-50
IB:αCHC
-180
αSCYL2
-100
IP:PTEN
αRab5
-30
αPTEN
-50

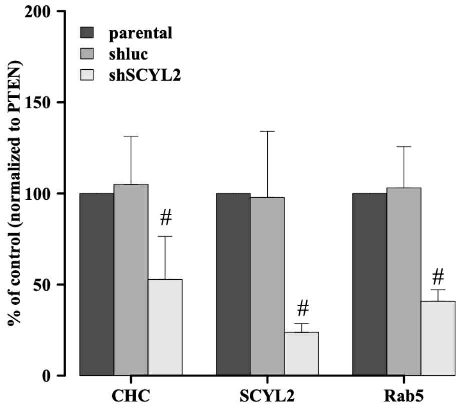
% of control (normalized to PTEN)
200
150
100
50
0
parental
shluc
shSCYL2
#
CHC
SCYL2
Rab5

K

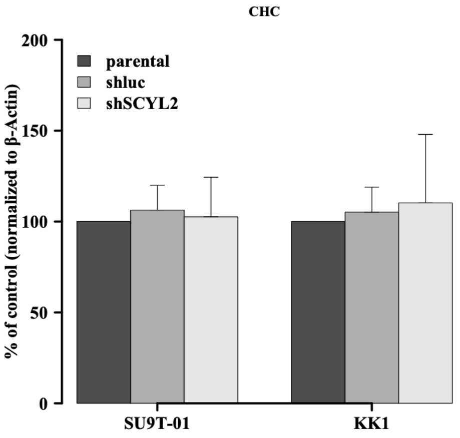
CHC
% of control (normalized to β-Actin)
200
150
100
50
0
parental
shluc
shSCYL2
SU9T-01
KK1

Supplementary Fig. 4.

A, A picture of Coomassie brilliant blue stained SDS-PAGE gel with co-immunoprecipitated proteins by control IgG or anti-SCYL2 antibody. Selected protein bands digested with Lys-C and Trypsin (1 to 7) were identified by MS analysis (**Supplementary Table 2**). Molecular weights are indicated in kDa.

B, Top 8 GO terms (biological process (BP), cell component (CC), and molecular function (MF). Only significantly represented GO terms were considered (adjusted p-value < 0.0001). Circle sizes represent the number of binding-proteins in each function, and bubble colors correspond to p values.

C, Cell lysates from KK1 were precipitated by anti-SCYL2 or anti-CHC antibody, and the precipitated proteins were immunoblotted with the indicated antibodies.

D, CHC or Rab5 (Alexa Fluor-488, green) and SCYL2 (Alexa Fluor-555, red) were detected in KK1 by immunofluorescent staining, and cell nuclei were stained with DAPI (blue). Scale bar, 5 µm. A region identified by the white box is further magnified to show the colocalization.

E, Quantification of colocalization of SCYL2 with CHC or Rab5 was carried out using Pearson's correlation coefficient from 20 representative images. The minimal value for significant colocalization is 0.5 (dotted line). The mean and SD are shown (n = 20).

F, Dot plot of relative gene expression levels of CHC in PBMCs (n = 3) and $CDAM1^{+}/CD7^{-}$ ATL patients (n = 3) (GSE55851). Data were showed as the mean and SD; #$p < 0.05$ versus PBMCs. Bot plot of relative gene expression levels of CHC in CD4 (n = 5) and acute ATL patients (n = 7) (GSE43017). Data were showed as the mean and SD; #$p < 0.05$ versus CD4.

G, qPCR analysis of CHC mRNA in CD4 (n = 5) and primary ATL cells (n = 5). Data were presented in dot plot, and showed as the mean and SD.

H, Cell Lysate from KK1 cells (parental, shluc, shCHC-1, and shCHC-2) was investigated using antibodies specific to each immunoblot. The results are representative of three independent experiments. Bar graphs show the quantification of relative band intensity normalized to β-actin. The mean and SD are shown ($n = 3$); #$p < 0.05$, versus parental.

I, Kinase activity of immunoprecipitates for 293T cells transfected with Flag-SCYL2 and/or HA-CHC expression vectors with each specific antibody was measured using an *in vitro* kinase assay with GST-PTEN as the substrate. The results are representative of three independent experiments. Bar graphs show the quantification of the relative band intensity normalized to PTEN. The mean and SD are shown ($n = 3$); #$p < 0.05$, versus mock.

J, Cell lysate form KK1 cells (parental, shluc, and shSCYL2) was investigated using specific antibodies in immunoblots. PTEN immunoprecipitated from KK1 cells were analyzed by immunoblotting with the indicated antibodies. The results are representative of three independent experiments. Bar graphs show the quantification of the relative band intensity normalized to immunoprecipitated PTEN. The mean and SD are shown ($n = 3$); #$p < 0.05$, versus parental.

K, Bar graphs show the quantification of the relative band intensity of CHC normalized to β-actin in SU9T-01 and KK1 cells. The mean and SD are shown (n = 3).

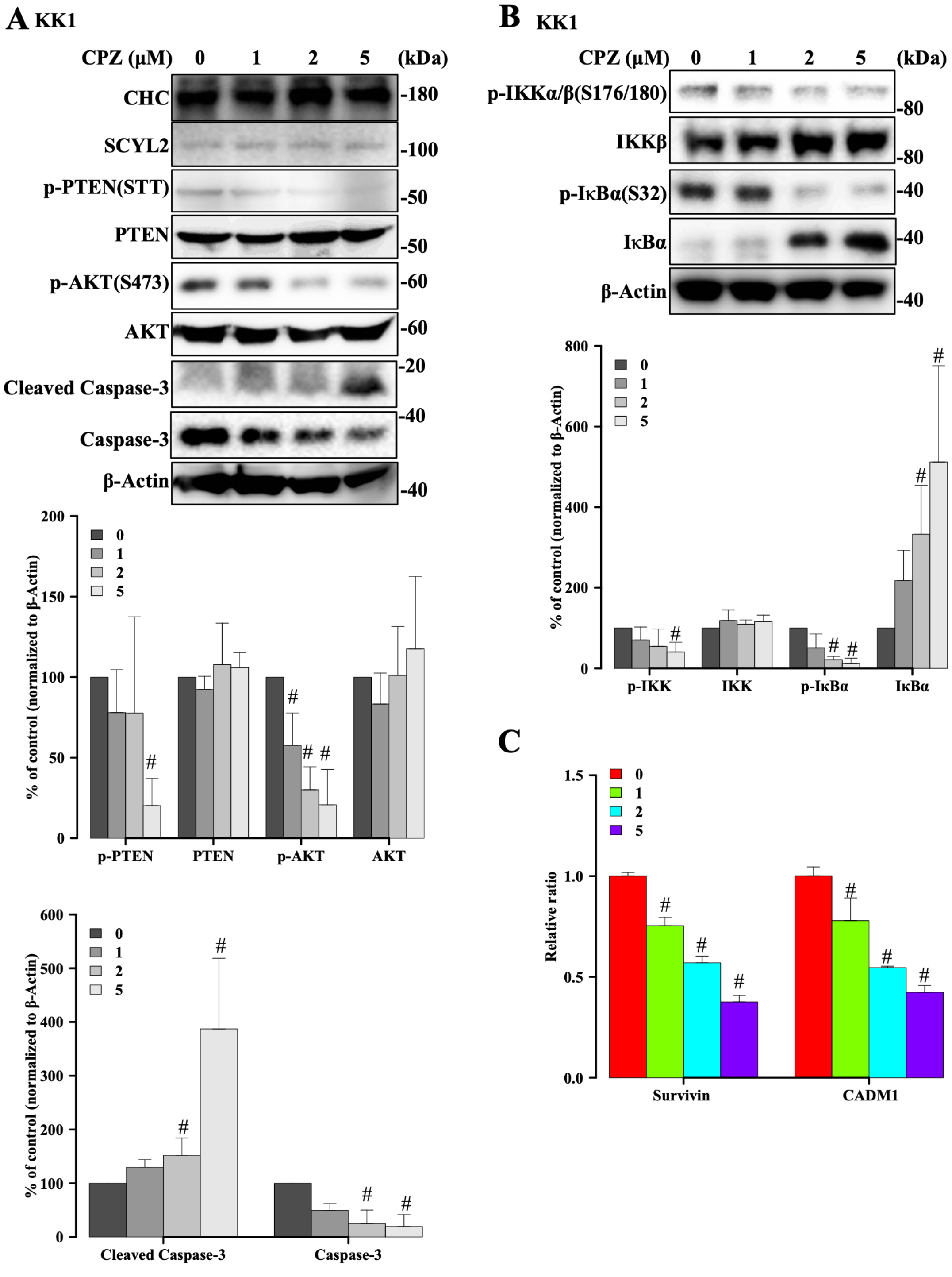
A KK1
CPZ (μM) 0 1 2 5 (kDa)
CHC -180
SCYL2 -100
p-PTEN(STT) -50
PTEN -50
p-AKT(S473) -60
AKT -60
Cleaved Caspase-3 -20
Caspase-3 -40
β-Actin -40
% of control (normalized to β-Actin)
p-PTEN PTEN p-AKT AKT
Cleaved Caspase-3 Caspase-3
B KK1
CPZ (μM) 0 1 2 5 (kDa)
p-IKKα/β(S176/180) -80
IKKβ -80
p-IκBα(S32) -40
IκBα -40
β-Actin -40
% of control (normalized to β-Actin)
p-IKK IKK p-IκBα IκBα
C
Relative ratio
Survivin CADM1

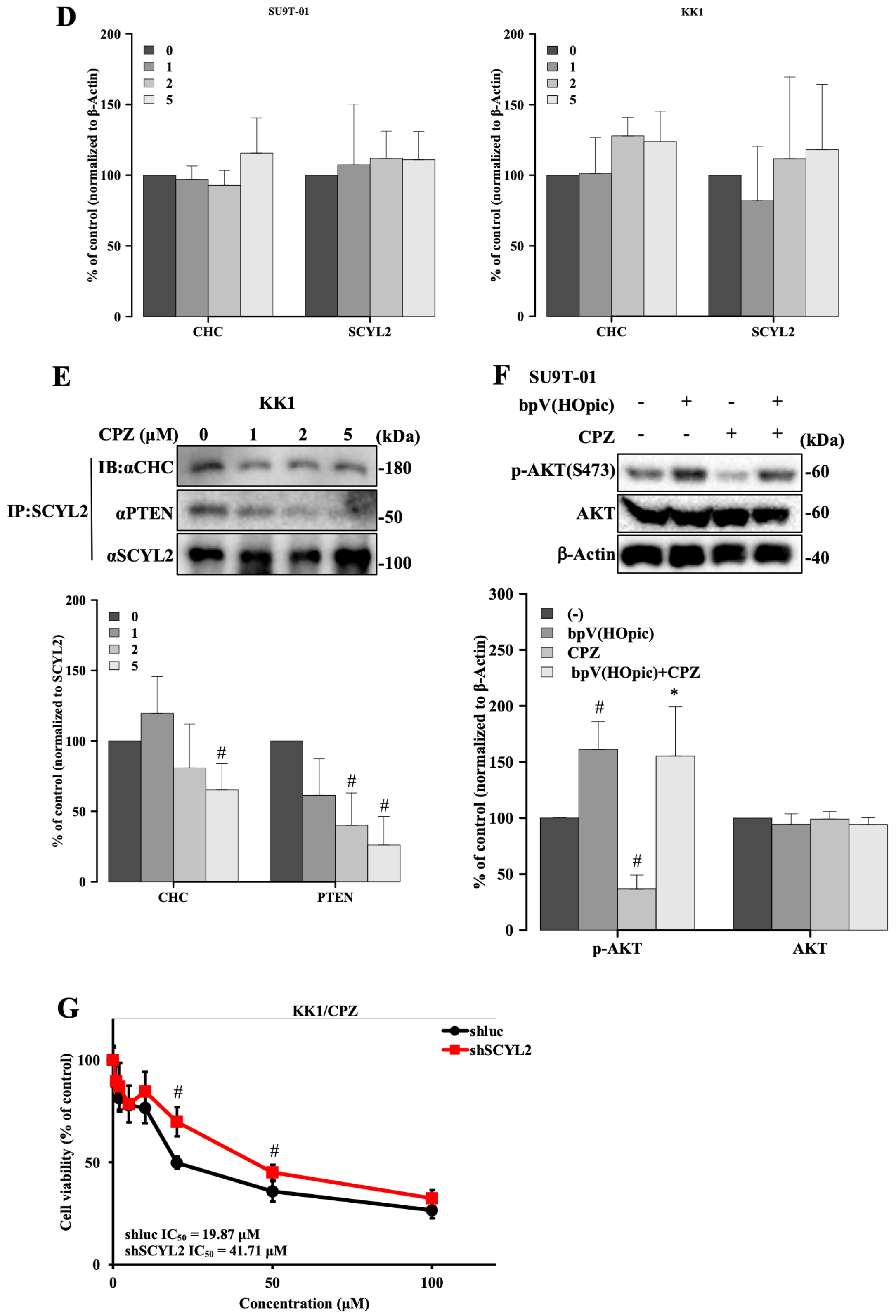
D
SU9T-01
KK1
% of control (normalized to β-Actin)
0
1
2
5
CHC
SCYL2
E
KK1
CPZ (μM) 0 1 2 5 (kDa)
IP:SCYL2
IB:αCHC -180
αPTEN -50
αSCYL2 -100
% of control (normalized to SCYL2)
CHC
PTEN
#
F
SU9T-01
bpV(HOpic) - + - +
CPZ - - + + (kDa)
p-AKT(S473) -60
AKT -60
β-Actin -40
% of control (normalized to β-Actin)
(-)
bpV(HOpic)
CPZ
bpV(HOpic)+CPZ
p-AKT
AKT
#
*
G
KK1/CPZ
shluc
shSCYL2
Cell viability (% of control)
Concentration (μM)
shluc IC50 = 19.87 μM
shSCYL2 IC50 = 41.71 μM

Supplementary Fig. 5.

A, KK1 cells were treated with the indicated doses of CPZ for 24 h, and determined by immunoblot analysis. The results are representative of three independent experiments. Bar graphs show the quantification of the relative band intensity normalized to β-actin. The mean and SD are shown (n = 3); *$p < 0.05$ versus 0

B, Cell lysate from KK1 cells treated with the indicated doses of CPZ for 24 h was subjected to western blot analysis of NF-κB pathway. The results are representative of three independent experiments. Bar graphs show the quantification of relative band intensity normalized to β-actin. The mean and SD are shown (n = 3); #$p < 0.05$, versus 0.

C, Quantitative PCR analysis of NF-κB target genes in KK1 cells treated with the indicated doses of CPZ for 24 h. The mean and SD are shown (n = 4); #$p < 0.05$, versus 0.

D, Bar graphs show the quantification of the relative band intensity of CHC and SCYL2 normalized to β-actin in SU9T-01 and KK1 cells treated with or without CPZ. The mean and SD are shown (n = 3).

E, SCYL2 was immunoprecipitated from KK1 cells treated with the indicated doses of CPZ for 24 h; it was then analyzed via immunoblotting using the indicated antibodies. Bar graphs show the quantification of the relative band intensity normalized to immunoprecipitated SCYL2. The mean and SD are shown (n = 3); #$p < 0.05$ versus 0.

F, SU9T-01 cells were treated with or without the indicated doses of bpV(HOpic) and CPZ for 24 h, and determined by immunoblot analysis. The results are representative of three independent experiments. Bar graphs show the quantification of the relative band intensity normalized to β-actin. The mean and SD are shown (n = 3); #$p < 0.05$ versus (-); *$p < 0.05$ versus CPZ.

G, Cell viability and IC50 were determined using Cell Counting Kit-8 after treatment with 0-100 μM CPZ for 24 h in KK1 cells (shluc and shSCYL2). The mean and SD are shown ($n = 4$); #$p < 0.05$, versus shluc.

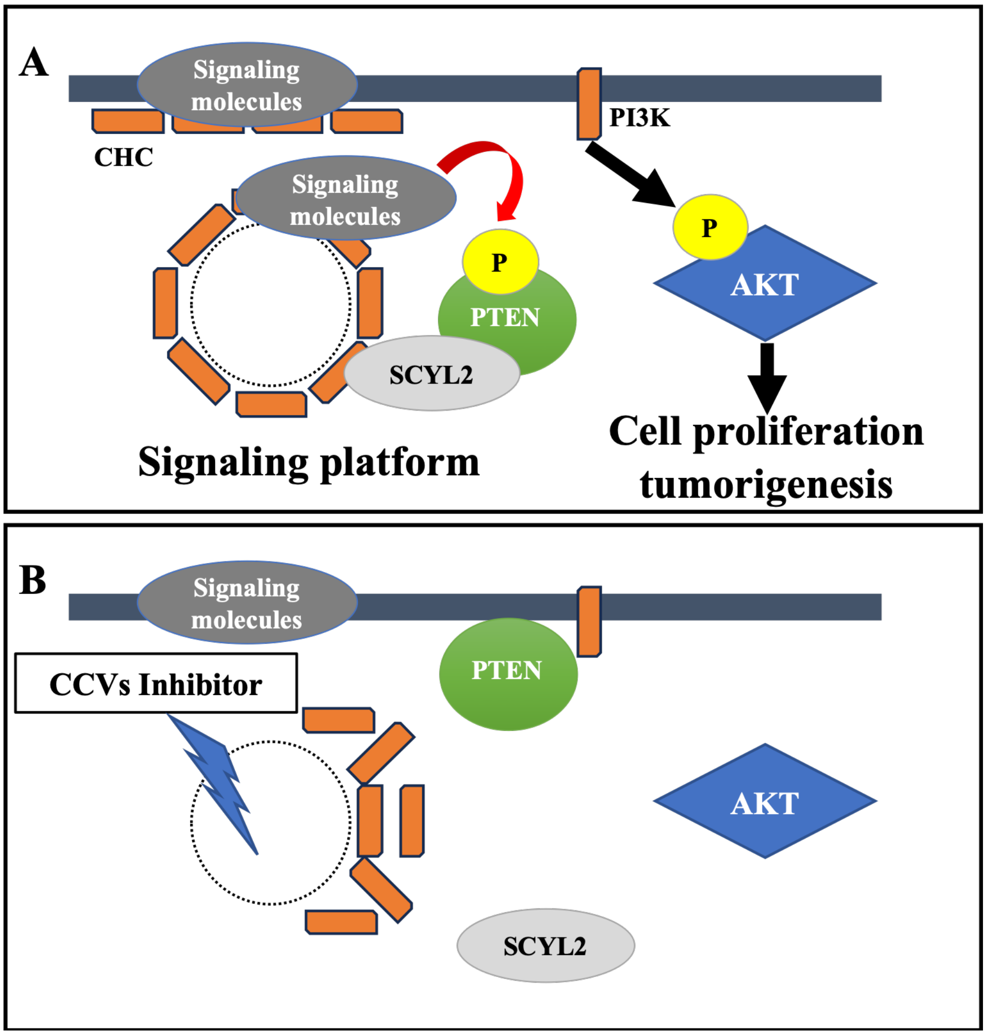

Supplementary Fig. 6.

A, Clathrin-coated vesicles (CCVs) complex with SCYL2, PTEN, and signaling molecules, and it can signal from vesicles. PTEN is phosphorylated by SCYL2/CHC-associated complex followed by the activation of PI3K/AKT signaling pathway in ATL, resulting in cell proliferation and tumorigenesis.

B, The suppression of SCYL2 expression and/or treatment with CCVs inhibitor has become a feasible and effective strategy for promoting cancer sensitivity in ATL.
